# Child development and distance learning in the age of COVID-19

**DOI:** 10.1007/s11150-022-09606-w

**Published:** 2022-04-05

**Authors:** Hugues Champeaux, Lucia Mangiavacchi, Francesca Marchetta, Luca Piccoli

**Affiliations:** 1 CRED, University of Namur, Namur, Belgium; 2 Department of Political Sciences, University of Perugia, Perugia, Italy; 3 IZA, Institute of Labor Economics, Bonn, Germany; 4 CERDI—Université Clermont Auvergne—CNRS, Clermont-Ferrand, France; 5 Department of Sociology and Social Research, University of Trento, Trento, Italy

**Keywords:** Children’s education, Education inequality, Distance learning, Emotional wellbeing, COVID-19, I24, J13, J24

## Abstract

School closures, forcibly brought about by the COVID-19 crisis in many countries, have impacted children’s lives and their learning processes. The heterogeneous implementation of distance learning solutions is likely to bring a substantial increase in education inequality, with long term consequences. The present study uses data from a survey collected during Spring 2020 lockdown in France and Italy to analyze parents’ evaluations of their children’s home schooling process and emotional well-being at time of school closure, and the role played by different distance learning methods in shaping these perceptions. While Italian parents have a generally worse judgment of the effects of the lockdown on their children, the use of interactive distance learning methods appears to significantly attenuate their negative perception. This is particularly true for older pupils. French parents rather perceive that interactive methods are effective in mitigating learning losses and psychological distress only for their secondary school children. In both countries, further heterogeneity analysis reveal that parents perceive younger children and boys to suffer more during this period.

## Introduction

The COVID-19 crisis in Spring 2020 forced many countries around the world to close schools for a prolonged period of time, and teaching has been moved online on an unprecedented scale.^1^ Even within the same countries or regions, teachers and schools have adopted different learning solutions, in order to guarantee continuity in teaching and learning. As a consequence, inequality in human capital development is likely to increase for the affected cohorts of children.

In this paper, we describe the perceptions of Italian and French parents about the effects of the spring 2020 lockdown on the learning progresses and emotional status of their children, aged 3 to 16. We aim to determine whether distance learning solutions adopted by teachers have been effective in mitigating the negative perceived effects of the lockdown.

School closures during the lockdown obliged teachers to suddenly adopt distance learning strategies, but often without receiving clear guidelines from their superiors. Schools and teachers were thus free to choose from a large typology of methods, which differ in the degree of interaction. This provides a useful setting to study the impact of different distance learning solutions on young students. During the Spring 2020 lockdown, we collected original data on a sample of Italian and French families, with specific information about each child. This data allows us to perform child fixed effect regressions to analyze the difference in parents’ evaluation of their children’s home learning and emotional status when live classes or chats have been implemented, compared to less interactive methods, such as sharing materials or videos. The study of parental perceptions about their children educational progress and emotional wellbeing can be particularly relevant as these perceptions, rather than true characteristics, drive their investment in human capital (Attanasio, 2015; Bergman, 2021; Dizon-Ross, 2019; Kinsler & Pavan, 2021; Nicoletti & Tonei, 2020). Moreover, early education literature showed strong correlations between parental perceptions on children’s academic performances and objective performances (Maguin & Loeber, 1996; Weine et al., 1990). On the other hand, the psychological literature routinely studies children’s psychological well-being through their parents’ perceptions. This has been also done during the COVID-19 lockdown, for instance by Pisano et al. (2020), Orgilés et al. (2020), and Stassart et al. (2021). For all these reasons, identifying those children who, according to their parents, have suffered more during the lockdown, might help policymakers to target interventions aimed at containing the surge of educational inequality.

The cross-country focus on France and Italy is noteworthy since both countries were hugely affected by COVID-19 in 2020 and their school systems are mostly public. This implies that the analysis would not be severely confounded by children’s enrollment in private schools that are more likely to have better educational technologies, as shown for the UK (Andrew et al., 2020).^2^ At the same time, the comparison between France and Italy is interesting because their educational systems differ in terms of both policy priority and results (Woessmann, 2016). They also differ in terms of the duration of school closure: Italy started on March 4, 2020, keeping schools closed until the end of the academic year; French schools closed on March 17, 2020 and gradually reopened starting from May 10 on a voluntary basis. There have been also important differences in how distance learning solutions have been provided during the crisis.

The paper contributes to the literature on educational technology and distance learning, evaluating parents’ perceptions about the effectiveness of different distance learning approaches. Before the COVID-19 pandemic, the economic literature related to educational technology focused on college students, who were the subjects of a number of experiments (Bettinger et al., 2017; Coates et al., 2004; Pellizzari et al., 2019; Xu & Jaggars, 2013), which showed mixed evidence on the effects of online classes on achievement compared to traditional lectures. In regard to the differences between alternative online learning solutions, Figlio et al. (2013) analyzed the difference between live classes and watching videos with the same lectures on the internet in a experimental settings and found that live-only instruction is slightly better than internet instruction. The recent widespread use of educational technology due to school closures, pushed this literature to expand. Orlov et al. (2021) and Kofoed et al. (2021) find that, compared to traditional classes, online lecture reduce college students’ achievements by about 0.2 standard deviations. On the other hand, Angrist et al. (2020) find that even low-tech solutions, such as SMS sent to parents, can improve achievements with respect to having no interaction, but to a small extent, 0.12 standard deviations. Finally, Carlana and La Ferrara (2021) shows that an experimental intervention of live tutoring in Italian lower secondary schools improved students achievements by 0.26 standard deviations. We add to this literature showing that interactive distance methods are perceived as effective in containing the learning loss implied by not attending classes in presence. We further show that parents’ perceptions on the emotional status of their children are also sensitive to the type of pedagogical methodologies used by teachers.

We find that during the lockdown, on average, Italian parents were more worried about their children’s home learning process and emotional well-being with respect to their French counterparts. Parents perceived their younger children (and to a lesser extent boys) to suffer more from the lockdown, both in terms of learning progresses and emotional status. Children attending secondary schools also experienced significant losses in terms of learning progress when they could not attend online classes, and this is particularly evident in France, where almost 30 percent of them did not benefit from interactive distance learning methods. In general, the use of interactive methods seems to attenuate the negative effect on learning and emotional status that parents attributed to school closure. We notice that this effect is again stronger for Italy.

The rest of the paper is organized as follows. “Education systems and the pandemic in France and Italy” section describes the institutional settings, focusing on education systems and the management of distance learning during school closure in the two countries. “Data” and “Model” sections describe the data and the estimation strategy, respectively. “Results” section presents the results of the empirical analysis and “Conclusions” section concludes.

## Education systems and the pandemic in France and Italy

### Institutional setting

The organization and governance of the educational system, combined with family background, are able to explain a large part of international differences in student achievement (Woessmann, 2016). Family background and institutions are quite likely to also shape the educational penalty that children of different countries may have suffered from the school closure period. It is not merely that differently organized schools may have offered distance learning solutions that are likely heterogeneous in quality, but also that pupils who have been trained to be self-directed in their academic work may have experienced lower losses.

French and Italian school systems share some similarities but also have significant institutional differences. Table A.1 shows that they are both largely public systems^3^ characterized by compulsory education until 16 years of age. Both countries have four levels of education and teachers have about the same starting salary (about 30 K dollars PPP for kindergarten and primary education, and about 32.5 K for secondary education). Despite being apparently similar, the French system achieves better results. According to the 2018 OECD PISA report, French scores are higher than Italian in all subjects: reading, mathematics, and science. In addition, French schools achieve higher attendance rates at all levels, but particularly at early ages.^4^

Italian primary and lower secondary students go to school more days during the year (200 vs 162, about 23.5% more), but school days are much more concentrated, as summer holidays last 4/5 weeks more in Italy. French classes are larger by more than 4 students on average and French teachers have more pupils at all levels. Public expenditure per student is larger in France (except for primary education) and overall public expenditure on education/GDP is almost 50% larger in France. Part of the difference is reflected on schools’ IT endowment, as France has a much larger number of PC, laptops or tablets per 100 students, while Italian schools are slightly better equipped with interactive whiteboards. Finally, French schools have much younger teachers: primary school teachers under 30 make up 12% of the total versus 1% of Italy, while the share of teachers aged 50 or more are 22% of the total versus 56% of Italy.

There are, however, other characteristics of the school organization that are not evident from official statistics, but that are likely to be relevant for students’ achievement: for instance, in Italian schools most children in primary and lower secondary school maintain the same teachers for the entire duration of the school level, while in France this typically does not happen, with most teachers changing every year. In addition, classmates and classrooms change from one year to the next, and, for older children, even during the day. The lack of teachers turnover in the Italian school system is likely to generate strong bonds between students and teachers, which can be good in a perspective of social interaction, but can make children learning process more teacher-dependent, making students less autonomous in their educational career. Thus, the sudden break of such relationships due to schools closure might have had particularly negative effects on Italian children.

### Education during the pandemic

In 2020, the COVID-19 pandemic hit early both in Italy and France, with the first confirmed cases occurring in the last days of January. The contagion evolution forced both governments to act with nationwide restrictive measures. In Italy, all schools closed on March 4 (some regions closed schools a couple of weeks earlier), while the French government followed early on, closing schools on March 16. By March 17, both countries had already implemented home confinement measures and by March 23 both countries had already issued travel limitations to citizens. These measures stayed in place until May 11, when both counties started removing limitations. France gradually reopened schools at the end of the lockdown, with full re-opening set on June 22. In Italy the same happened only after the summer holidays, on September 14 for most regions.^5^

During the closure of school buildings, educational activities were maintained by the French and Italian governments. As the pandemic was not anticipated, schools and teachers from both countries benefited from some degree of freedom regarding the implementation of distance learning methods. In Italy, The Ministry of Education provided some guidelines indicating the software platforms that could be used, but schools had almost total freedom in deciding if and how to implement distance learning solutions. In France, the Ministry of Education decreed “pedagogical continuity” for the pupils early on, providing official chatrooms and educational platforms, but, as with Italy, teachers were not obliged to use them, and instead were free to decide what type of learning methods to offer to their students.^6^ In addition, children differed in terms of IT equipment availability,^7^ in terms of parental investment (which may depend on the parents’ level of education and working status during lockdown) and on the types of distance learning solutions they benefited from during lockdown. All these factors likely generated highly heterogeneous impacts of schools closures on children’s learning achievements and emotional status.

At time of writing, large scale data are still being processed and it is difficult to have a precise idea of the impact that school closures had on pupils’ academic achievement. Some studies tried to anticipate such results. For instance, Blaskó et al. (2021) use pre-covid data to simulate how a generalized closure would exacerbate educational inequality. Other studies used smaller scale data to provide an early assessment: Grewenig et al. (2021) find that low-achieving college students are paying a larger toll, while Rodríguez-Planas (2021) find that parental socio-economic status is important, with an opposite effect for high- and low-achieving university students.

As administrative data is becoming available, more insights on the overall impact of school closure is starting to emerge. For instance, the 2021 INVALSI report^8^ show that Italian primary school children managed to keep an average score similar to 2019, but secondary school students obtained significantly worse results. The share of students that did not obtain “adequate results” with respect to the national indications provided by the Ministry of Education increased by 5 percentage points for lower secondary schools and by 9 percentage points for high school, both in language and mathematics. In addition there has been substantial regional heterogeneity, with southern regions performing even worse, further increasing the north-south educational divide. In France, national evaluations established a significant drop in the performance in mathematics and in French for 1st grade primary school pupils enrolled in both private and public schools, with reading and writing skills being notably affected (DEPP, 2020). Surprisingly, pupils in the 1st grade secondary school had better scores in 2020 than in 2019, while in both primary and secondary schools, the gap between pupils in the poorest areas and the others increased during the period. Interestingly, 68% of the secondary school teachers declared that pupils satisfactorily learnt and became more autonomous during the schools closure.

## Data

We use original data from two surveys, specifically designed to study the effects of the lockdown on Italian and French families and their components, which we collected through an online questionnaire. Surveys were jointly developed with an European team of researchers. Similar surveys were also disseminated in Spain (Lidia Farré and Libertad Gonzales), Germany (Christiane Schwieren) and Austria (Doris Weichselbaum). The French and Italian surveys added a specific section on children. The anonymous questionnaires were disseminated through advertising campaigns on the main social networks, such as Facebook and Twitter, targeted to working age individual and to households with children. Participation was on a voluntary basis and no rewards were offered upon completion of the questionnaire.^9^ We started to disseminate the surveys on April 7 in Italy and on April 21 in France. Both surveys were available until the end of the outbreak, on May 10. The final sample used in the paper is composed of 3,769 Italian children and 3,183 French children, respectively from 2,455 and 1,838 families. As the participation in the surveys was voluntary with no sampling strategy, we cannot claim representativity of the populations of reference at national levels. For Italy, thanks to the relevant sample size and the ability to reach all the regions and different socio-economic groups, the geographical and family type distributions are in line with the national statistics reported by ISTAT (see Table A.2, Panel A). The only notable exceptions are for the South of Italy, which is slightly under-represented, and for the share of mono-parental households, which is strongly under-represented. The situation is similar for France: the sample is relatively well balanced at the geographical level (excepted for the Paris area), while single parents are still under-represented (see Table A.2, Panel B). In both countries, the educational level of the interviewed families is substantially higher with respect to national statistics, as well as the probability of being employed.^10^

The surveys included basic information on the respondents’ and their partners’ personal characteristics including gender, age, location of residence, highest level of education, marital status, and parental status. They also collected detailed current and retrospective information on the respondents’ and their partners’ labor market participation, division of household tasks and children’s activities (Champeaux & Marchetta, 2021; Mangiavacchi et al., 2021).^11^ Basic information on all children living in the household (i.e., age, gender, school level) were asked to parents, as well as questions on children’s time use before and after school closures. The surveys also asked parents their subjective opinions on the child’s learning improvement during lockdown, and on her/his emotional status. Finally, it contains information about the distance learning methods offered to each child during lockdown and on IT equipment availability.

Table 1 shows that children are balanced on gender in both countries and are a little older in France (the average age is 9.6) than in Italy (the average age is 8.3). Reflecting the difference in fertility rates between the two countries, we note that 81 percent of children in France and 74 percent in Italy have siblings.^12^ All children of our sample are gathered in three schooling categories: kindergarten, primary and secondary schools. For France, 24.9 percent of the children surveyed were in kindergarten, 42.5 percent in primary and 32.6 percent in secondary. For Italy, 27.7 percent of the children were in kindergarten, 47.1 percent in primary and 25.2 percent in secondary.

**Table 1:** Descriptive statistics for France and Italy

	France			Italy				
	*N*	Mean	sd	*N*	Mean	sd	Max	Min
Outcomes								
Learning progress	3183	−3.391	2.284	3769	−5.138	2.614	0	−9
Emotional status	3183	−0.306	0.833	3769	−0.670	0.957	2	−2
Schooling level								
Kindergarten	3183	0.249	0.432	3769	0.277	0.447	1	0
Primary	3183	0.425	0.494	3769	0.471	0.499	1	0
Secondary	3183	0.326	0.469	3769	0.252	0.434	1	0
Distance learning methods								
No contents	3183	0.008	0.088	3769	0.112	0.316	1	0
Online courses	3183	0.403	0.491	3769	0.635	0.482	1	0
Homework	3183	0.589	0.492	3769	0.253	0.435	1	0
Characteristics								
Gender (=1 for Girl)	3183	0.490	0.500	3769	0.491	0.500	1	0
Age	3183	9.607	3.758	3769	8.342	3.505	18	2
Having siblings	3183	0.808	0.394	3769	0.740	0.439	1	0
University (Parents)	3183	0.569	0.495	3769	0.586	0.493	1	0
At least one parent at home	3183	0.912	0.283	3769	0.858	0.401	1	0
Facing an economic shock	3183	0.404	0.491	3769	0.264	0.441	1	0

Descriptive statistics are based on data collected through online survey during the Covid-19 pandemic. Data collection was from 21 April to 11 May for France (1838 families), and from 7 April to 11 May for Italy (2455 families). “Learning progress” and “Emotional status” are variables based on the parental perceptions of the living conditions of their children before and during the lockdown. “Having siblings” is a dummy equal to one when the child has at least one sister or brother. “University” is a dummy equal to one when at least one parent is graduated from the University. For France, 2101 children have at least one out of two parents staying at home during lockdown; 802 have both parents (or the parent who stayed at home) teleworking; 280 have their both parents outside. For Italy, 3025 children have at least one out of two parents staying at home during lockdown; 210 have both parents (or the parent who stayed at home) teleworking; 534 have their both parents outside. “Facing an economic shock” is a dummy equal to one when the household experienced an earning reduction or if at least one member lost his/her job

Almost 60 percent of children in the two samples have at least one parent with a university degree. Furthermore, at least one parent did not have to work out of home during the lockdown for 80 percent of Italian children and 91 percent of French ones. Concerning the working situation, our data show that a non negligible part of children have parents who were hit by an economic shock: 26.4 percent of Italian and 40 percent of French children have at least one parent who lost her/his job, had an activity suspension or an earning reduction during the lockdown.^13^

In both countries, parents perceived that, during the lockdown period, their children have allocated a significant part of their time that was previously devoted to school to passive screen time. Parents report that time spent watching TV or on the internet (videos, socials) doubled in both countries, increasing from 1 to 2 hours on average for French children and from 1.5 to 3 hours on average for the Italian ones (see Tables A.3 and A.4). At the same time, according to parents, French children increased the daily reading time. A similar increase is observed for Italian pre-school children.^14^

### Learning achievements and emotional status

With the closure of schools, children’s living conditions harshly changed during the lockdown. Directly linked to the closure, a concern for parents was to appreciate the effect on their learning achievements. For each child, our surveys ask parents to evaluate their learning progress using a 10-point scale. Here, we note 0 when the child progressed at the same pace as when she/he was attending classes at school (the maximum) and -9 when the child did not progressed at all.^15^ In Table 1, the descriptive statistics show that French parents had a better judgment of children learning than Italian ones. This may be due to different, but not alternative, explanations. First, on April 13, the French President announced that schools would be reopened starting from May 11. This may have reassured French parents about the temporary nature of school closures, while no statements was given at that time by the Italian Prime Minister, clearly indicating a more than likely reopening after the summer vacation (which actually happened). Second, the level of parental stress could have been higher in Italy at the time of the survey because of the larger number of cases and deaths.^16^ Individuals living in the most affected areas may have worse perceptions also because more likely to be directly affected by COVID-19, for instance someone in their family or network could be sick.^17^ Finally, the difference could depend on the type of school inputs children received before the lockdown: the French system, especially in kindergarten and primary schools, seems to prepares children to be more independent and more flexible to changes (see “Institutional setting” section), so French children may have adapted better to homeschooling during the lockdown.

With schools closure and the lockdown, children’ social life was also heavily affected. The COVID-19 outbreak increased the stress and burden on parents and the social isolation of children from their peers and teachers. This situation could have affected the socio-emotional skills of children, such as their mental health, wellbeing, and behavior. The risk of an increase in socio-emotional problems may be higher for those living in low educated and the poorest households, who have lower socio-emotional skills in normal periods (Attanasio et al., 2020). Boys are also more at risk since they are more likely to experience behavioral issues than girls (Autor et al., 2016; Bertrand & Pan, 2013; Chetty et al., 2016), as well as all adolescents. On the other hand, positive interactions between parents and children can improve socio-emotional skills (Moroni et al., 2019). The survey asked parents to report the evolution of their children’s emotional status^18^ in a −2 to 2 scale.^19^ The data show that French parents had slightly better perceptions of their children emotional status during the school’s closure than the Italian parents (Table 1). Again, this can be related to both contingent and structural differences between the two countries.

In our empirical analysis, both variables, learning achievements and emotional status, are standardized with mean 0 and standard deviation 1 for each country.

### Distance learning methods

Upon closure in March 2020, in both countries teachers had to put in place distance learning activities, even if they were not prepared in the slightest for such a task. The Ministries of Education provided some guidance and offered some software platforms that could be used, but schools and teachers had almost total freedom in deciding if and how to implement distance learning activities. The unexpectedness of this event caused a quick but extremely heterogeneous response (DEPP, 2020).

Presuming such diversities in distance learning methods adoption, the survey asked parents to report the distance learning activities that were being offered to their children. Questionnaires differ in terms of available items in the two countries. The Italian questionnaire asked parents if teachers (i) offered live full or partial online lectures; (ii) shared only educational material, by mail or other digital platforms; (iii) did not offer any distance learning activity. In the French questionnaire, more details were asked about the distance learning methods offered by teachers. Parents could select multiple options for distance learning. Six options were provided: (i) online lectures; (ii) material provided by emails without interactive content; (iii) pedagogical videos from their teachers; (iv) pedagogical videos from other teachers; (v) chat room with other pupils and the teachers; (vi) no material provided by the teachers. Besides the latter option, the other choices were not independent and, for example, individuals could select both the “chat room” and the “pedagogical videos” options. Gathering those who only received videos and homework without interactive contents for the French respondents, we obtain the corresponding category (ii) for the Italian respondents. Attending chat room and online courses can also be gathered to be similar to category (i) in the Italian survey. Therefore, we obtain three main groups of children that differ according to the distance learning methods used during the lockdown: pupils who both followed online lectures and received materials by email, pupils who were connected with the teachers only by emails or internet platforms and did homework, and pupils who did not receive any contents from teachers and had no relationship with them.

**Fig. 1:**
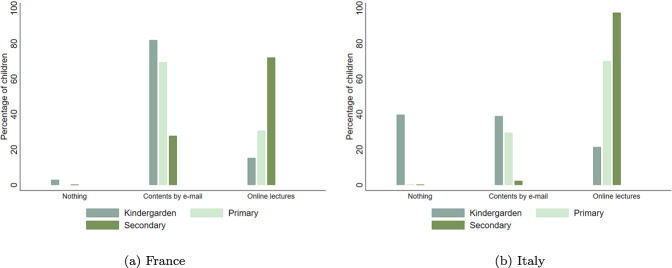
Distance learning methods during the lockdown. The category “Contents by email” gathers children who only received pedagogical material without interaction with their teachers. “Online Lectures” is a category with children receiving both materials by emails and also interactive contents with the class and the teacher. **a** The sample of French children. **b** The sample of Italian children

Figure 1 shows substantial differences between the two countries and across school levels. Only 40.3 percent of children in our French sample followed online courses and interactive lessons with their teachers. Italian children benefited at 63.5 percent from live online classes. Consequently, we note the inverse distribution for pupils having only homework without interactive contents during the lockdown between France and Italy, respectively 58.9 percent and 25.3 percent. Disentangling by school level also shows interesting discrepancies. At kindergarten, 39.6 percent of Italian children were not involved in any activity, while this was the case only for 2.9 percent of French children. Even if online lectures are unsurprisingly less common for young children, 21.3 percent of the Italian kindergarten pupils followed such lessons while they were 15.2 percent in France. In both countries, less than 0.5 percent of primary and secondary school children did not received any content by their teachers. Online classes were offered to 70 percent of Italian primary school students and to 30.6 percent of French ones. Concerning secondary school students, while in Italy almost all of them attended online classes (97.4 percent), the percentage of online lectures for France stands at 72.1 percent. Furthermore, most children in our sample had access to IT equipment, such computers, tablets or smartphones. For France, less than 0.5% of primary and secondary school children did not have access to IT equipment while the figure rises to almost 7% in Italy.^20^

**Fig. 2:**
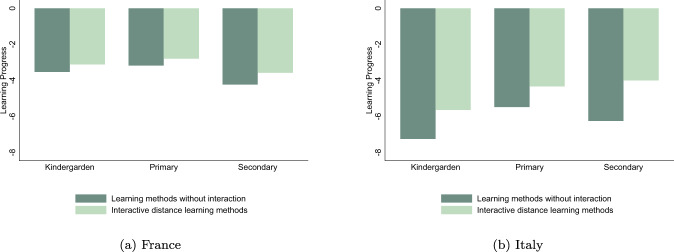
Differences in learning progress across distant learning methods. **a** The sample of French children. **b** The sample of Italian children

**Fig. 3:**
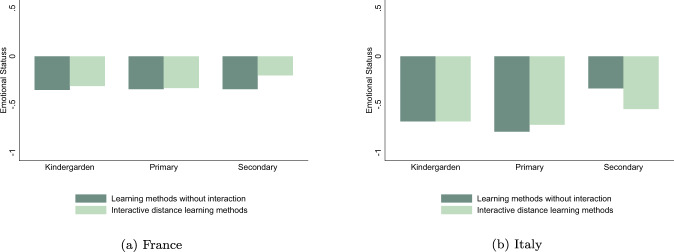
Differences in emotional status across distant learning methods. **a** The sample of French children. **b** The sample of Italian children

The type of distance learning activities proposed by teachers seems to drive parents’ evaluation of their children learning progress during the lockdown, especially for older children. Figure 2 shows that, for both Italy and France, parental judgment was better when children were able to follow interactive lectures and the difference grows larger with school levels. In Italy, the overall judgment of parents was particularly low for all school levels when no interactive classes were offered and parental evaluation substantially improves with the availability of online classes. The relationship between distance learning methodologies and parental perceptions on children’s emotional status is less straightforward. While for French parents the availability of interactive lectures is correlated to a better perception of their mental health, in particular for the secondary school children, no clear pattern emerge for Italian parents (Fig. 3).^21^ These correlations induced us to further explore the relationship between distance learning methodologies and parental perceptions of children learning and emotional well-being.

## Model

In this paper, we investigate the relationship between the distance learning methods followed by the pupils and their wellbeing during the pandemic, in terms of learning progress and emotional status. More specifically, we analyze whether interactive learning solutions, when offered, mitigated the negative effects of school’s closure and to which extent. In order to compare results across countries, we standardize the learning progress and emotional wellbeing changes at country level with a mean of zero and a standard deviation of one. Even if these variable were assessed at the time of the pandemic, we retrieve past information as the pretended value without lockdown (the base is 0 for both our outcomes). Interpreting it as a variation with the lockdown allow us to perform fixed effects model as:

1 $${Y}_{it}=\theta Lockdow{n}_{t}+\beta OnlineCourse{s}_{it}\times Lockdow{n}_{t}+\gamma {X}_{it}+{u}_{i}+{e}_{it}$$

considering that *t* takes only values 0 and 1, the model’s parameters of interest can be estimated by a first difference model:

2 $${{\Delta }}{Y}_{it}={Y}_{i0}-{Y}_{i1}=\theta +\beta {{\Delta }}OnlineCourse{s}_{it}+\gamma {{\Delta }}{X}_{it}+{{\Delta }}{e}_{it}$$

where *Y*_*it*_ is the selected outcome (learning progress or emotional status) for the child *i* at the time *t*. *Lockdown*_*t*_ is a temporal dummy equal to one for the period during the lockdown. *OnlineCourses*_*it*_ is a dummy equal to one if the pupil had at least one interactive learning method with the teacher during school closure. The coefficient *β* of the interaction between *Lockdown*_*t*_ and *OnlineCourses*_*it*_ captures the differential effects of the lockdown for children attending online lessons compared to the others. As we can observe in Fig. 1, very few children did not benefit of any pedagogical contents during the lockdown and most of them are in kindergarten. Therefore, in our main estimates gathering all children regardless of their school level, we withdraw children who had not pedagogical contents during lockdown. The *Lockdown*_*t*_ coefficient, *θ*, consequently captures the effect of having educational contents without interaction, named *Homework*. In the tables reporting estimation results, *θ* coefficients are presented under *Homework* ⋅ *Lockdown*. We also provide overall effects of the lockdown using the same specification without the interacted terms.

*X*_*it*_ is a set of child-specific time-varying regressors. Here, to account for the possible effect of different allocation of children’s time (Fiorini & Keane, 2014), we include the time spent in front of passive screens and reading, in hours per day, before and during the lockdown.^22^

To account for the possible increase in parental time input, especially relevant for young children development (Del Boca et al., 2014; Del Bono et al., 2016), we also include two dummies indicating whether the mother and father were actually working for any amount of time (including smartworking or teleworking) before and during the lockdown.^23^*u*_*i*_ represents child fixed effects and *e*_*it*_ is the idiosyncratic error. Standard errors are clustered at regional level. Because of the differences in the original questions on distance learning and the importance to show differences between countries, models are separately estimated for Italy and for France.

Identification issues can be raised in our models. We check selection bias by verifying that pre-lockdown observable characteristics of sample children who received online classes do not strictly differ from those who only benefited from homework. In Tables A.5 and A.6, we show that there does not exist a clear pattern of selection between the two different groups of children even if some differences remain significant. Second, while having a first difference model solves systematic perception bias of parents, the recall error related with the pre-lockdown measure might still differ according to unobservable parents’ characteristics that vary over time or that could affect children in a different way over time.

## Results

### Learning achievements and distance learning methods

We first present estimates using as dependent variable parent’s perception of their children’s learning progress, gathering all children from different school levels. In Table 2, columns 1 and 2 show results for France, columns 3 and 4 for Italy. As outcomes are standardized at country level, our results can be compared across estimates. In columns 1 and 3, we observe that results are quite similar in terms of magnitude for France and Italy, with a negative and significant global effect of the lockdown on learning progress. Interestingly, we note discrepancies between Italian and French children when we take into account the heterogeneity in terms of learning solutions adopted by their teachers during the pandemic. These results are reported in columns 2 and 4. For Italy, the coefficient of the interaction between “Online Lectures” and “Lockdown” is positive and significant, meaning that the negative effect of the school closure was attenuated by the use of interactive methods. Thus, according to Italian parents, children who attended online lectures had better learning progress than those who only benefited from homework without interactive contents. We do not observe the same for France, where the negative perceived effect of school closure on learning progress does not seem to be influenced by the choice of a particular distance learning method. Italian parents were more worried about their children’ learning when they did not follow online lectures, while this was not the case in France.

**Table 2:** Effects of distance learning methods (DLM) on learning progress

	France		Italy	
		DLM		DLM
	(1)	(2)	(3)	(4)
Lockdown	−1.435***		−1.455***	
	(0.049)		(0.058)	
Homework (Ref.)		−1.462***		−1.805***
⋅ Lockdown		(0.051)		(0.082)
Online Lectures		0.069		0.482***
⋅ Lockdown		(0.048)		(0.055)
*N*	6316	6316	6692	6692
Children (*N*)	3158	3158	3346	3346
Within *R*^2^	0.698	0.698	0.793	0.809

All results were estimated using first-difference models on original datasets from Italian and French 2020 Covid-19 online surveys. “Lockdown” is a dummy variable equal to one for the period during the school closures and zero before. Parental evaluation of the children’s learning progress is defined in the “Learning achievements and emotional status” section and standardized in the estimates with a mean of zero and a standard error of one. “Homework” is a dummy equal to one for children benefited from pedagogical contents without interactions with their teachers during the lockdown. “Online Lectures” is a dummy equal to one for children benefited from online interactive lessons during the lockdown. In these estimates, all retained children followed either Homework, or Online Lessons. Therefore, “*Homework* *⋅* *Lockdown*” is purely similar to the “*Lockdown*” term, constituent of the interactive variable “*OnlineLectures* *⋅* *Lockdown*”. Coefficient in front of the interactive variable “*OnlineLectures* *⋅* *Lockdown*” must be interpreted as a differential effect from the category of reference, “*Homework* *⋅* *Lockdown*”. Each specification controls for a set of time-variant covariates as working status of the mother and the father, children’s time-use in reading and in front of passive screen. Each specification also controls for child individual fixed effects. Standards errors in parentheses are clustered at region level

***,**,*denote significance at 1%, 5%, 10% level, respectively

The high diversity of learning methods across school levels, shown in Fig. 1, needs to be accounted for in order to discriminate potentially heterogeneous effects. In Table 3 for France and in Table 4 for Italy, we first present baseline estimates (columns 1 and 2) and then report estimates of children’s learning progress for each school level. Parents of children in kindergarten are those who were the most concerned by the absence of any pedagogical contents during the lockdown. This situation was relatively more widespread in Italy than in France, affecting almost 40 per cent of Italian children in kindergarten and only 3 per cent in France. For this school level, we thus create two subsamples, one including children who did not receive any educational contents from their teachers (columns 3 and 4) and another one excluding them, similarly as the samples used in the baseline models (columns 5 and 6). In both France and Italy, children who were categorized as “No Contents” made lower learning progresses than all others. While French children in kindergarten attending online lectures did not present any difference with respect to those who only had homework, Italian children having interactive learning lessons performed better that those without interactive contents.

**Table 3:** Effects of distance learning methods (DLM) on learning progress [France]

	Baseline		Kindergarten^a^		Kindergarten		Primary		Secondary	
		DLM		DLM		DLM		DLM		DLM
	(1)	(2)	(3)	(4)	(5)	(6)	(7)	(8)	(9)	(10)
Lockdown	−1.435***		−1.433***		−1.416***		−1.330***		−1.593***	
	(0.049)		(0.085)		(0.084)		(0.064)		(0.080)	
Homework (Ref.)		−1.462***		−1.419***		−1.429***		−1.380***		−1.770***
⋅ Lockdown		(0.051)		(0.080)		(0.082)		(0.061)		(0.090)
Online lectures		0.069		0.073		0.071		0.155*		0.248***
⋅ Lockdown		(0.048)		(0.141)		(0.141)		(0.073)		(0.075)
No contents				−0.726*						
⋅ Lockdown				(0.398)						
*N*	6316	6316	1582	1582	1536	1536	2708	2708	2072	2072
Children (*N*)	3158	3158	791	791	768	768	1354	1354	1036	1036
Within *R*^2^	0.698	0.698	0.686	0.691	0.691	0.691	0.670	0.672	0.744	0.748

All results were estimated using first-difference models on original datasets from Italian and French 2020 Covid-19 online surveys. “Lockdown” is a dummy variable equal to one for the period during the school closures and zero before. Parental evaluation of the children’s learning progress is defined in the “Learning achievements and emotional status” section and standardized in the estimates with a mean of zero and a standard error of one. “Homework” is a dummy equal to one for children benefited from pedagogical contents without interactions with their teachers during the lockdown. “Online Lectures” is a dummy equal to one for children benefited from online interactive lessons during the lockdown. Except for columns (3) and (4), all retained children followed either Homework, or Online Lessons. Therefore, “*Homework* *⋅* *Lockdown*” is purely similar to the “*Lockdown*” term, constituent of the interactive variable “*OnlineLectures* *⋅* *Lockdown*”. Coefficient in front of the interactive variable “*OnlineLectures* *⋅* *Lockdown*” must be interpreted as a differential effect from the category of reference, “*Homework* *⋅* *Lockdown*”. Each specification controls for a set of time-variant covariates as working status of the mother and the father, children’s time-use in reading and in front of passive screen. Each specification also controls for child individual fixed effects. Standards errors in parentheses are clustered at region level

***,**,*denote significance at 1%, 5%, 10% level, respectively

^a^Sample for all children in Kindergarten including those who did not benefit from any pedagogical contents during the lockdown, gathered under the dummy variable named “No Contents”. In this specification, the coefficient in front of “NoContents ⋅ Lockdown” must be interpreted as a differential effect from the category of reference “Homework ⋅ Lockdown”

**Table 4:** Effects of distance learning methods (DLM) on learning progress [Italy]

	Baseline		Kindergarten^a^		Kindergarten		Primary		Secondary	
		DLM		DLM		DLM		DLM		DLM
	(1)	(2)	(3)	(4)	(5)	(6)	(7)	(8)	(9)	(10)
Lockdown	−1.455***		−2.120***		−1.884***		−1.417***		−1.233***	
	(0.058)		(0.068)		(0.088)		(0.059)		(0.071)	
Homework (Ref.)		−1.805***		−1.957***		−1.960***		−1.670***		−1.995***
⋅ Lockdown		(0.082)		(0.090)		(0.093)		(0.083)		(0.224)
Online lectures		0.482***		0.209**		0.212**		0.353***		0.781***
⋅ Lockdown		(0.055)		(0.076)		(0.077)		(0.076)		(0.209)
No contents				−0.591***						
⋅ Lockdown				(0.099)						
*N*	6692	6692	2086	2086	1258	1258	3538	3538	1896	1896
Children (*N*)	3346	3346	1043	1043	629	629	1769	1769	948	948
Within *R*^2^	0.793	0.809	0.889	0.910	0.862	0.864	0.796	0.806	0.776	0.782

All results were estimated using first-difference models on original datasets from Italian and French 2020 Covid-19 online surveys. “Lockdown” is a dummy variable equal to one for the period during the school closures and zero before. Parental evaluation of the children’s learning progress is defined in the “Learning achievements and emotional status” section and standardized in the estimates with a mean of zero and a standard error of one. “Homework” is a dummy equal to one for children benefited from pedagogical contents without interactions with their teachers during the lockdown. “Online Lectures” is a dummy equal to one for children benefited from online interactive lessons during the lockdown. Except for columns (3) and (4), all retained children followed either Homework, or Online Lessons. Therefore, “*Homework* *⋅* *Lockdown*” is purely similar to the “*Lockdown*” term, constituent of the interactive variable “*OnlineLectures* *⋅* *Lockdown*”. Coefficient in front of the interactive variable “*OnlineLectures* *⋅* *Lockdown*” must be interpreted as a differential effect from the category of reference, “*Homework* *⋅* *Lockdown*”. Each specification controls for a set of time-variant covariates as working status of the mother and the father, children’s time-use in reading and in front of passive screen. Each specification also controls for child individual fixed effects. Standards Errors in parentheses are clustered at region level

***,**,*denote significance at 1%, 5%, 10% level, respectively

^a^Sample for all children in Kindergarten including those who did not benefit from any pedagogical contents during the lockdown, gathered under the dummy variable named “No Contents”. In this specification, the coefficient in front of “NoContents ⋅ Lockdown” must be interpreted as a differential effect from the category of reference “Homework ⋅ Lockdown”

Interestingly, this pattern is similar for all school levels in Italy, where parents had a better perception of learning achievement when their children followed online lectures across all levels. Furthermore, there is an increase in magnitude from kindergarten to secondary school: higher is the school level, better is the parental perceptions of the learning progress when their children attended online lessons. For France, even if we do not observe a significant effect on average neither in the baseline, nor for children in kindergarten, the subsample analysis unveils that French parents give better evaluation of learning achievement of their primary and, in particular, secondary school pupils when they benefited from interactive learning methods. Therefore, for both primary and secondary levels, our estimates indicate that, according to parents’ perceptions, interactive lectures are more advantageous for educational progress than non-interactive methods.

On average, Italian parents are more worried regardless of the child’s level when online courses are not offered. This may suggest that they consider their children less independent than French ones, and, as a consequence, they are reassured when their children have a closer contact with their teachers. This difference across countries could also be explained by the perspectives of re-opening the French schools in late May 2020 (see “Learning achievements and emotional status” section), that could have reassured French parents, even in the absence of interactive distance learning methods.^24^

In what follows, we explore the heterogeneity in response to the lockdown and to the distance learning methods used by teachers, in different sub-populations. We first split the samples by gender, following the stream of literature that suggests that boys may be more vulnerable to school and home environment (Autor et al., 2016, 2019; Bertrand & Pan, 2013; Chetty et al., 2016). As parents’ expectations of the learning autonomy of their children could be driven by gendered stereotypes, we estimate our baseline models on subsamples of young boys and girls. Results are shown in columns 3 to 6 of Table A.7 for France, and of Table A.8 for Italy. For comparison, baseline estimates are reproduced in columns 1 and 2 of these tables. Italian parents are more worried, on average, for boys than for girls, meaning that, with the schools closure, they considered girls being more able to adapt themselves to the situation than boys. Furthermore, in terms of distance learning methods, the online lectures coefficient is only slightly larger for girls than for boys, suggesting that parents perceived a similar impact. We do not obtain similar results for France. First, parents’ perception of the lockdown on learning progress is similar across gender, on average. Second, while we do not observe any average effect of interactive lessons, the coefficient for online lectures is positive and significant only for girls. This suggests that the average effect we observe hides gender heterogeneity of responses to the distance learning methods.

We then look at the heterogeneous results with respect to some family characteristics. Having siblings at home could affect the way parents perceive the learning progress because children could help each other with homework or, at the opposite, could disturb each others and may have conflicting time schedule for online lessons. Columns 7 to 10 of Tables A.7 and A.8 show the results on the subsamples of children without and with siblings. We find small differences between the two subsamples: the lockdown effect on learning seems slightly worse for Italian children in the absence of siblings, while French parents consider that their children benefit from online lessons only when they have siblings. Finally, more educated parents are likely to be more comfortable in taking care of their children’s education. When we split the samples between children with at least one parents highly educated (having a university degree) and children without high educated parents (columns 11 to 14 of Tables A.7 and A.8), we observe that, in France, parents’ education level reduces the negative expectation of the lockdown on children’s learning progress. Education, however, does not change the perception of distance learning methods. We observe the opposite for Italy, where parents perception of the lockdown does not differ in the two groups, but more educated parents perceive a higher positive effect of online lectures.^25^

### Emotional status and distance learning methods

We now investigate the link between lockdown, distance learning solutions and children’s emotional status, using the same empirical framework adopted for analyzing learning progress in “Learning achievements and distance learning methods” section. Table 5 reports estimates on the full samples for France (columns 1 and 2) and for Italy (columns 3 and 4). On average, Italian parents perceived a stronger impact of the lockdown on children’s emotional wellbeing than French parents. The “Lockdown” coefficient for the French sample (column 1) is about half the Italian one (column 3).^26^ Concerning the heterogeneous effect by distance learning methods, children from both countries experienced better emotional status when they followed online lectures (column 2 and 4), according to their parents’ judgment. The coefficient is positive and significant, although only at 10%, meaning that in comparison to children having only homework (the category of reference), having interactive courses reduce the overall negative effect of the lockdown.

**Table 5:** Effects of distance learning methods on emotional status

	France		Italy	
	All	DLM	All	DLM
	(1)	(2)	(3)	(4)
Lockdown	−0.379***		−0.796***	
	(0.066)		(0.075)	
Homework (Ref.)		−0.441***		−0.884***
⋅ Lockdown		(0.068)		(0.085)
Online lectures		0.158*		0.122*
⋅ Lockdown		(0.074)		(0.067)
*N*	6316	6316	6692	6692
Children (*N*)	3158	3158	3346	3346
Within *R*^2^	0.129	0.132	0.344	0.345

All results were estimated using first-difference models on original datasets from Italian and French 2020 Covid-19 online surveys. “Lockdown” is a dummy variable equal to one for the period during the school closures and zero before. Parental evaluation of the children’s mental health is defined in the “Learning achievements and emotional status” section and standardized in the estimates with a mean of zero and a standard error of one. “Homework” is a dummy equal to one for children benefited from pedagogical contents without interactions with their teachers during the lockdown. “Online Lectures” is a dummy equal to one for children benefited from online interactive lessons during the lockdown. In these estimates, all retained children followed either Homework, or Online Lessons. Therefore, “*Homework* *⋅* *Lockdown*” is purely similar to the “*Lockdown*” term, constituent of the interactive variable “*OnlineLectures* *⋅* *Lockdown*”. Coefficient in front of the interactive variable “*OnlineLectures* *⋅* *Lockdown*” must be interpretated as a differential effect from the category of reference, “*Homework* *⋅* *Lockdown*”. Each specification controls for a set of time-variant covariates as working status of the mother and the father, children’s time-use in reading and in front of passive screen. Each specification also controls for child individual fixed effects. Standards errors in parentheses are clustered at region level

***,**,*denote significance at 1%, 5%, 10% level, respectively

Sub-sample analysis by school level are presented in Tables 6 and 7, respectively for France and Italy. First of all, in both countries parents are more worried about the negative psychological effect of the lockdown on their younger children (in kindergarten and in primary school) than for they secondary-school pupils.^27^ We also notice that, for France, online lectures are only significant and positive for secondary school pupils. The positive average effect is thus driven by this category of children. French parents did not perceive any beneficial effect of interactive learning methods for the mental health of children in kindergarten or in primary school. For Italy, even if we observe a positive and significant average effect of online courses on emotional status, the sub-sample analysis does not reveal a specific category driving this effect.

**Table 6:** Effects of distance learning methods (DLM) on emotional status [France]

	Baseline		Kindergarten^a^		Kindergarten		Primary		Secondary	
		DLM		DLM		DLM		DLM		DLM
	(1)	(2)	(3)	(4)	(5)	(6)	(7)	(8)	(9)	(10)
Lockdown	−0.379***		−0.398**		−0.397***		−0.439***		−0.272**	
	(0.066)		(0.132)		(0.129)		(0.101)		(0.112)	
Homework (Ref.)		−0.441***		−0.395**		−0.396**		−0.442***		−0.425***
⋅ Lockdown		(0.068)		(0.140)		(0.137)		(0.101)		(0.094)
Online lectures		0.158*		−0.003		−0.002		0.010		0.214**
⋅ Lockdown		(0.074)		(0.188)		(0.189)		(0.111)		(0.092)
No Contents				−0.065						
⋅ Lockdown				(0.270)						
*N*	6316	6316	1582	1582	1536	1536	2708	2708	2072	2072
Children (*N*)	3158	3158	791	791	768	768	1354	1354	1036	1036
Within *R*^2^	0.129	0.131	0.170	0.170	0.171	0.171	0.156	0.156	0.083	0.087

All results were estimated using first-difference models on original datasets from Italian and French 2020 Covid-19 online surveys. “Lockdown” is a dummy variable equal to one for the period during the school closures and zero before. Parental evaluation of the children’s mental health is defined in the “Learning achievements and emotional status” section and standardized in the estimates with a mean of zero and a standard error of one. “Homework” is a dummy equal to one for children benefited from pedagogical contents without interactions with their teachers during the lockdown. “Online Lectures” is a dummy equal to one for children benefited from online interactive lessons during the lockdown. Except for columns (3) and (4), all retained children followed either Homework, or Online Lessons. Therefore, “*Homework* *⋅* *Lockdown*” is purely similar to the “*Lockdown*” term, constituent of the interactive variable “*OnlineLectures* *⋅* *Lockdown*”. Coefficient in front of the interactive variable “*OnlineLectures* *⋅* *Lockdown*” must be interpretated as a differential effect from the category of reference, “*Homework* *⋅* *Lockdown*”. Each specification controls for a set of time-variant covariates as working status of the mother and the father, children’s time-use in reading and in front of passive screen. Each specification also controls for child individual fixed effects. Standards errors in parentheses are clustered at region level

***,**,*denote significance at 1%, 5%, 10% level, respectively

^a^Sample for all children in Kindergarten including those who did not benefit from any pedagogical contents during the lockdown, gathered under the dummy variable named “No Contents”. In this specification, the coefficient in front of “NoContents ⋅ Lockdown” must be interpreted as a differential effect from the category of reference “Homework ⋅ Lockdown”

**Table 7:** Effects of distance learning methods (DLM) on emotional status [Italy]

	Baseline		Kindergarten^a^		Kindergarten		Primary		Secondary	
		DLM		DLM		DLM		DLM		DLM
	(1)	(2)	(3)	(4)	(5)	(6)	(7)	(8)	(9)	(10)
Lockdown	−0.796***		−0.760***		−0.855***		−0.830***		−0.657***	
	(0.075)		(0.129)		(0.179)		(0.111)		(0.094)	
Homework (Ref.)		−0.884***		−0.768***		−0.851***		−0.887***		−0.606
⋅ Lockdown		(0.085)		(0.170)		(0.187)		(0.117)		(0.462)
Online Lectures		0.122*		0.007		−0.011		0.079		−0.052
⋅ Lockdown		(0.067)		(0.108)		(0.117)		(0.072)		(0.448)
No Contents				0.018						
⋅ Lockdown				(0.147)						
*N*	6692	6692	2086	2086	1258	1258	3538	3538	1896	1896
Children (*N*)	3346	3346	1043	1043	629	629	1769	1769	948	948
Within *R*^2^	0.344	0.345	0.346	0.346	0.348	0.348	0.404	0.404	0.248	0.248

All results were estimated using first-difference models on original datasets from Italian and French 2020 Covid-19 online surveys. “Lockdown” is a dummy variable equal to one for the period during the school closures and zero before. Parental evaluation of the children’s mental health is defined in the “Learning achievements and emotional status” section and standardized in the estimates with a mean of zero and a standard error of one. “Homework” is a dummy equal to one for children benefited from pedagogical contents without interactions with their teachers during the lockdown. “Online Lectures” is a dummy equal to one for children benefited from online interactive lessons during the lockdown. Except for columns (3) and (4), all retained children followed either Homework, or Online Lessons. Therefore, “*Homework* *⋅* *Lockdown*” is purely similar to the “*Lockdown*” term, constituent of the interactive variable “*OnlineLectures* *⋅* *Lockdown*”. Coefficient in front of the interactive variable “*OnlineLectures* *⋅* *Lockdown*” must be interpretated as a differential effect from the category of reference, “*Homework* *⋅* *Lockdown*”. Each specification controls for a set of time-variant covariates as working status of the mother and the father, children’s time-use in reading and in front of passive screen. Each specification also controls for child individual fixed effects. Standards errors in parentheses are clustered at region level

***,**,*denote significance at 1%, 5%, 10% level, respectively

^a^Sample for all children in Kindergarten including those who did not benefit from any pedagogical contents during the lockdown, gathered under the dummy variable named “No Contents”. In this specification, the coefficient in front of “NoContents ⋅ Lockdown” must be interpreted as a differential effect from the category of reference “Homework ⋅ Lockdown

Similar to what done in the “Learning achievements and distance learning methods” section, we finally explore the existence of possible heterogeneous results according to some children and parents’ characteristics. Estimates are presented in Tables A.9 and A.10, respectively for France and Italy.

In terms of gender disparities, we find results similar to schooling achievement. Italian parents are more worried, on average, for the emotional status of boys during the lockdown. This can be explained by gender stereotypes (i.e., parents might perceive girls as tolerating better to stay at home than boys), or could reflect the fact that, objectively, boys have suffered more during the lockdown. For France, we note that, parents’ perception with respect to their male children are better when they attend online lessons, while this is not true for girls. Again, this might be interpreted with gender stereotypes: as boys need more social interaction than girls, parents credit emotional wellbeing advantage to the interactive distance learning methods for them. This is in line with previous findings, for instance by Bertrand and Pan (2013), Chetty et al. (2016), and Autor et al. (2019).

Having siblings is associated to a better mental health during the school closure according to Italian parents, and to a worse one according to French parents. In both countries, parents indicate that following on line courses attenuate the negative effect of lockdown only in presence of other children at home.^28^ Finally, lower educated italian and french parents are more worried about their children mental health.^29^

## Conclusions

School closures, forcibly caused by the COVID-19 crisis in many countries, modified children’s learning processes with likely consequences in terms of achievements and educational inequality. In addition, the lack of peer interactions could have affected the socio-emotional skills of children.

This paper contributes to the recent literature trying to evaluate the effect of different distance learning methods on children’s learning achievements at time of the school closure (e.g., Angrist et al., 2020; Carlana & La Ferrara, 2021). Our main objective is to determine whether distance learning solutions adopted by teachers mitigated the negative effects of the lockdown by analyzing parents’ perceptions. In particular, we analyze how the Spring 2020 lockdown has affected children’s emotional well-being and home learning processes at different school levels in France and Italy. To this aim, we collected data on a large sample of families with children in April and early May 2020.

We show important differences in the distance learning solutions adopted by teachers and schools during the lockdown both across countries and across school levels. In particular, the share of students that were offered interactive learning methods is larger in Italy and for higher grades students.

We also note that both French and Italian parents were particularly worried by their children’s home learning processes. For children in pre-primary and primary school levels, Italian parents had significantly worse perceptions than French parents. Our estimates show that the learning progress has been particularly hampered for very young children (aged 3–6), especially for the ones who did not receive any distance learning support from their teachers (i.e., 40% of them in Italy versus only 3% in France). As early-age inputs are crucial for the children’s cognitive and non-cognitive development,^30^ developing adequate programs to recover the additional learning loss they suffered should be a priority for educational systems and policymakers. Children attending secondary schools also experienced important losses in terms of learning achievements when they could not attend online classes, and this is particularly evident in France, where it was the case for almost 30% of them.

More generally, our findings suggest that attending online classes played a role in reducing the negative impact of the lockdown on the home learning process, and that the compensating effect of interactive methods was stronger for older children. This could reflect the fact that it can be difficult for teachers to interact remotely with young children, especially at kindergarten and first years of primary education. Moreover, parents may find that it is more demanding to support their pre-school children learning progress as the teaching methods for this age group are less standardized and demand more creative skills.

In terms of emotional well-being, Italian children suffered more than French ones. Their parents reported a worse emotional status for younger children in both countries, while online classes seem to have attenuated the social capital losses of secondary-school pupils during the lockdown. Indeed, much of the negative effect of the lockdown on children’s emotional status may be due to their very limited interactions with peers. For older children, this reduction in personal interaction may have been partially compensated by virtual interactions during online classes, which could have mitigated the negative effect of the lockdown on their emotional status.

At time of writing, the sanitary crisis is still ongoing, with classes and even entire schools still moving to distance learning for limited periods of time. As shown by our results, current technologies for online classes are perceived by parents of secondary school children as being quite effective in partially compensating the learning loss determined by distance learning. On the other hand, online classes seem less effective for younger children, arguing that governments should be particularly concerned about keeping them at school for as long as possible. At the same, governments should invest more on the teachers’ training to help them better ensuring the continuity of learning when schools or classes are closed. In addition, given that objective evaluations may differ from parents’ perceptions,^31^ schools should implement actions to precisely inform parents about the actual academic loss their children experienced during school closures, and potential actions to let them recover. Because parents’ perceptions of academic achievements will drive their future human capital investment decisions (Bergman, 2021; Dizon-Ross, 2019; Kinsler & Pavan, 2021), avoiding this mismatch would help containing the educational inequality rise that is currently emerging.

Our analysis has some limitations. First, similar to other studies based on online surveys, digitally deprived people are excluded from the analysis. Second, our study is not based on representative samples of the Italian and French populations. In particular, high educated parents are over-represented in our data. Finally, our results are specifically relevant in the short-medium run, within the current pandemic context and in the hopefully near post-pandemic administration. Nevertheless, we believe that they also have an external validity that goes beyond the current sanitary crisis. First, we are able to show that parents are responsive to the pedagogical methods proposed by teachers, since their perceptions on children learning and emotional status vary with the type of methods. Second, we show that interactive distance learning technologies could partially substitute live classes for secondary school children, at least for a limited period of time. On the other hand, younger children are more severely affected by the absence of in-person relationships with their teachers and classmates. Third, by comparing two countries with key differences in terms of the school system, we document that designing an educational system that encourage children’s learning independence can make a difference in case of service disruption.

## Supplementary information

Supplementary Tables A.1–A.10

French questionnaire_EN

Italian_questionnaire_EN

## Data Availability

Replication code is available upon request.

## Footnotes

1 According to UNESCO, up to 192 simultaneous country-wide closures had affected 91.2% of the world’s student population at the beginning of April 2020 (source: UNESCO Institute for Statistics Database, COVID-19 Impact on Education).

2 Andrew et al. (2020) collected data on children aged 4–15 between April 29 and May 12, 2020 in the UK and found large variations in home learning resources provided by schools and in parents’ ability to support home learning. They find that private schools are much more likely to offer online classes and, even in state schools, online classes are more likely to be offered to children living in richest families.

3 6.9% of pupils attend private schools in Italy, in France this is around 21.5%. In France, private schools are almost entirely publicly funded.

4 Since 2019, education has been compulsory from the age of 3 in France.

5 Only daycare services were allowed to work, under strict restrictions, starting from July 1.

6 For instance, the CNED platform ’Ma classe à la Maison’ was used only by about 24 per cent of lower secondary students (DEPP, 2020).

7 About 9 per cent of French school principals declared that all or most of their students had outdated, defective or unsuitable equipment (DEPP, 2020).

8 Available in Italian on the open data site invalsiopen.it.

9 An English translation of the questionnaires is available as online supplementary material.

10 This is possibly due to the design of the advertising campaign, which described the questionnaire as a scientific research project and may have discouraged less educated individuals to participate. Moreover, the sample does not include –by definition– households who do not have access to internet.

11 For many items, we asked the respondent to recall which was the situation just before the lockdown and which was the current situation, thus the situation during the lockdown.

12 Table A.2 also shows that more than half of the children live in two-children households (52 percent in France and 56 percent in Italy). As expected, the incidence of children living in families with three children is higher in France (23 percent vs 15 percent).

13 In both countries, subsidies to compensate for economic shocks were given to workers. In France, the “activité partielle” scheme was extensively used: employees with a contract were eligible to receive 70 per cent of their gross wage from their employers, who were then reimbursed by the state. In Italy, the government used the STW (Cassa Integrazione) scheme: 80 percent of gross wages of employees of firms who declared having been negatively affected by the crisis were paid by the state (payments were limited at 998 euros for wages up to 2,159 and at 1,199 euros for wages above that level). If formally the two systems were similar, it is possible that the French system was more efficient and more prompt to reply to workers’ needs (OECD, 2020).

14 Reading time is reported to be almost one hour in both countries before schools closure. In France it increased up to 1 hour and 20 minutes, 10 minutes more than in Italy. The importance of children’s time allocation for their cognitive development, and in particular reading and educational time, have been highlighted by Fiorini and Keane (2014) and Kalb and Van Ours (2014).

15 Because of the contingent situation, our source of information are parental perceptions rather than test scores or other measurable outcomes. Although we are not measuring actual learning achievements, the educational literature on the accuracy of parents’ perception of their children academic performance shows that there is a strong correlation between parents’ perceptions and actual achievements. This correlation is at times stronger than the ones with teachers’ perceptions (Maguin & Loeber, 1996; Weine et al., 1990).

16 According to the WHO Coronavirus Disease (COVID-19) Dashboard, by May 11, France had experienced 137,073 cases and 26,338 deaths and Italy 219,070 cases and 30,560 deaths.

17 A within-country test for this hypothesis, exploiting regional variations, suggests that this seems not the case (see “Emotional status and distance learning methods” section).

18 Again, one could doubt that parental perception of children emotional status may be affected by measurement error. However, the psychological literature has routinely used parental perceptions to study children’s psychological wellbeing, even during the lockdown (Orgilés et al., 2020; Pisano et al., 2020; Stassart et al., 2021).

19 The response items were: “it is much worse”, “it is slightly worse”, “remains stable”, “it is slightly better”, “it is much better”.

20 We might underestimate digitally deprived children from our analysis, though, as the data was collected through on an online survey.

21 Looking at the figure, it seems that Italian parents have a worse emotional status perception in secondary schools when their children followed interactive lectures. However, this result needs to be interpreted with caution because of the small size of the subsample of Italian secondary pupils who did not follow online lectures.

22 These variables are measured according to parent’s perceptions and not time diaries, thus they are possibly affected by measurement error. However, given that the timing of the retrospective information is short (between 1 and 2 months for all respondents) and that there is a very clear-cut change in life organization between before and during the lockdown, we believe that this issue has limited application in our analysis. A suggestive evidence to support this hypothesis is that the date of interview is not significantly correlated with children time use before the lockdown (correlation coefficient at −0.04 for passive screen and −0.03 for reading), nor with learning progress evaluation (correlation coefficient at − 0.01). We also verified that results are not affected when we remove time use variables of our specification.

23 This variable is thus not referring to a person who has a job contract or not, but rather if s/he had actually worked in the reference period. Several types of workers, especially in the public sector, may have been receiving a salary without working any hours during the lockdown.

24 The difference in the perceptions of Italian and French parents could be partially explained by the higher percentage of French children who are enrolled in private schools. Indeed, parents of children in private school might have, on average, better perceptions about their learning progress during the lockdown, possibly because they might be more confident about the ability of the school to follow their children. Because we did not collect information on the private or public nature of the school in which children are enrolled, we are unable to test for this possible channel. Note, however, that most private schools in France are publicly financed, their fees are very low, teachers are paid by the government, and their children do not perform better than the ones attending public schools (Fougère et al., 2017). It is thus unlikely that private schools were much better prepared to deal with distance learning solutions than public schools at the time of the Spring 2020 lockdown.

25 We also explore the heterogeneity according to other parents’ characteristics finding no relevant differences. In particular, we find that parents’ perceptions on children’s learning achievements do not change when the household was hit by an economic shock (loosing job, wage or earning loss due to an activity suspension or partial unemployment) or when parents were in teleworking. These additional results are available upon request.

26 This difference could be possibly explained by the higher number of COVID-19 cases and deaths. Indeed, individuals from most affected areas may have worse perceptions and have higher probability of being directly affected by COVID-19 because someone in their family or network is sick. We can test for this hypothesis at a country level, exploiting the differences across Italian (French) regions with respect to the number of deaths. For each country, we create an indicator for respondents located in regions heavily affected by the COVID-19 pandemic and interact this variable with lockdown to capture for specific effects related to the pandemic exposure in our sample. Results, available upon request, give no support for such an hypothesis.

27 In column 10 of Table 7, the coefficient of the interaction term between “Homework” and “Lockdown” is not significant, probably due to the narrow size of the children in this situation. It does not mean that this subgroup are not psychologically affected by the Covid19, as we can note an average negative effect on the subsample without interactive terms.

28 For France, the point estimate is larger when there are no siblings, but the coefficient is very imprecisely estimated in this sample, so we can not reject the null hypothesis that the coefficient is equal to zero.

29 As for the analysis of learning progress, we also find no significant heterogeneity results when other family and child characteristics are taken into account, including whether parents faced an economic shock or being teleworking during lockdown. Results are available upon request.

30 Heckman (2006) and Heckman et al. (2010) started a conspicuous stream of literature about estimating the long-run impact of early interventions on child development. Recent contributions include e.g., García et al. (2020) and Gertler et al. (2021).

31 For instance, INVALSI (2021) show that primary school students affected by school closure achieved similar results to the previous cohort, while parents were clearly particularly worried about their learning progress.
