# Supplementary material for: Child development and distance learning in the age of COVID-19
Source: Rev Econ Househ. 2022 Apr 5;20(3):659–85. doi: 10.1007/s11150-022-09606-w (PMC8982654; doi:10.1007/s11150-022-09606-w)
Supplement: Supplementary file 1 — Supplementary Tables A.1–A.10 [file 11150_2022_9606_MOESM1_ESM.pdf]

A Appendix

A.1 Supplementary Tables

Table A.1: Institutional comparison of the French and Italian educational systems

	France	Italy		France	Italy
Age of attendance			*Students enrolled in private institutions*		
Kindergarten	3-5	3-5	Kindergarten	13.3%	28.3%
Primary	6-10	6-10	Primary	14.9%	6.0%
Lower secondary	11-14	11-13	Lower secondary	22.1%	3.6%
Higher secondary	15-18	14-18	Higher secondary	29.0%	8.8%
School days per year			*Public expenditure per pupil (thous. US$ PPP)*		
Primary	162	200	Kindergarten	8.2	7.4
Lower secondary	162	200	Primary	7.6	8.0
Higher secondary	180	200	Lower secondary	10.6	8.9
Summer holidays			Higher secondary	14.1	9.4
weeks per year	8	12/13	*Starting salary of teachers (thous. US$ PPP)*		
Class size			Kindergarten	30.9	30.4
Primary	23.7	19.1	Primary	30.9	30.4
Secondary	25.2	21	Lower secondary	32.5	32.7
Pupils per teacher			Higher secondary	32.5	32.7
Kindergarten	23.3	12.2	*Attendance rate (% of the same age group)*		
Primary	19.2	11.5	Nursery	56.3%	29.7%
Lower secondary	14.4	11	Kindergarten	100.0%	93.9%
Higher secondary	11.4	10.4	Primary	99.7%	97.4%
IT endowment (computers/100 students)			Secondary	86.4%	84.8%
Kindergarten	6.3	-	*Primary school teachers by age class*		
Primary	14.4	6.7	Less than 30	12%	1%
Lower secondary	33.8	8.8	30-39	33%	11%
Higher secondary	43.9	6.8	40-49	34%	32%
IT endowment (interactive whiteboard/100 students)			50 or more	22%	56%
Kindergarten	0.4	-	*PISA scores*		
Primary	1.7	2.6	Reading	493	476
Lower secondary	1.8	2.7	Math	495	487
Higher secondary	1.3	2.0	Science	493	468
Public expenditure					
Share of total public expenditure	10.8%	8.9%			
Percentage of the GDP	3.7%	2.5%			

Source: OECD.stat, Eurydice, PISA-OECD, ISTAT and INSEE (last available year, most figures refer to 2017 or 2018)

Table A.2: Representativeness of the original sample

A) ITALY			B) FRANCE		
	Our sample	ISTAT		Our sample	INSEE
Family type[1,2]					
Single parents	7.8%	24.5%	Single parents	14.4%	22.8%
Couples with 1 child	44.0%	47.9%	Couples with 1 child	31.2%	44.8%
Couples with 2 children	46.8%	41.7%	Couples with 2 children	50.7%	38.7%
Couples with 3 or more children	9.1%	10.4%	Couples with 3 children	15.4%	12.7%
			Couples with 4 or more children	2.6%	3.8%
Parents characteristics[3,4]					
Mothers with university degree	58.2%	29.2%	Mothers with university degree	57.4%	30.9%
Fathers with university degree	36.9%	19.5%	Fathers with university degree	38.1%	26.4%
Mothers working	79.9%	62.8%	Mothers working	83.6%	77.5%
Fathers working	96.3%	84.9%	Fathers working	92.3%	86.6%
Geographical distribution[5,6]					
Piemonte	9.6%	6.6%	AURA	12.4%	27.9%
Valle d'Aosta	0.4%	0.2%	Bourgogne-Franche-Comté	4.3%	6.8%
Liguria	2.6%	2.2%	Bretagne	5.2%	4.5%
Lombardia	20.0%	16.1%	Centre-Val-de-Loire	3.9%	5.0%
Trentino Alto Adige	2.2%	1.7%	Corse	0.5%	0.2%
Veneto	9.2%	8.0%	Grand Est	8.5%	8.0%
Friuli Venezia Giulia	2.3%	1.9%	Hauts-de-France	9.2%	6.3%
Emilia Romagna	8.6%	6.9%	Île-de-France	18.9%	5.9%
Toscana	10.5%	6.0%	Normandie	5.1%	5.4%
Umbria	3.5%	1.4%	Nouvelle-Aquitaine	9.3%	7.6%
Marche	2.6%	2.6%	Occitanie	9.1%	9.9%
Lazio	9.1%	10.3%	Pays de la Loire	5.9%	4.4%
Abruzzo	1.5%	2.2%	PACA	7.8%	8.2%
Molise	0.4%	0.5%			
Campania	5.0%	10.6%			
Puglia	4.7%	7.1%			
Basilicata	0.5%	0.9%			
Calabria	1.3%	3.3%			
Sicilia	3.2%	8.8%			
Sardegna	2.8%	2.8%			

Notes: 1. ISTAT – Multipurpose Survey on Households: Aspects of Daily Life 2019. 2. INSEE – Census 2016 3. ISTAT – 2019 Labor force survey. 4. INSEE – 2020 Labor force survey 5. ISTAT – Resident Municipal Population on January 1 2019. 6. INSEE – Census 2016

A.2 Daily Activities before and during lockdown

Table A.3: Time-variant observables for France

	(1)		(2)		T-test
	Before l.d.		During l.d.		Difference
Variable	N	Mean/SE	N	Mean/SE	(1)-(2)
Daily hours of reading	3183	0.932	3183	1.355	-0.423***
		(0.012)		(0.017)	
Daily hours of screen	3183	1.058	3183	2.212	-1.154***
		(0.014)		(0.018)	
Working status of the mother	3183	0.845	3183	0.693	0.152***
		(0.006)		(0.008)	
Working status of the father	3183	0.825	3183	0.691	0.135***
		(0.007)		(0.008)	

Descriptive statistics are based on data collected through online survey during the Covid19 pandemic. Data collection was from 21 April to 11 May for France, and from 7 April to 11 May for Italy. "Daily hours of reading" is the number of hours spent in reading or listening relatives reading. "Daily hours of screen" is the number of hours spent in front of passive screen as TV, Internet, social network etc. "Working status" is a dummy equal to one when the household member declares (or is declared) working in the period (including smartworking during the lockdown).
The T-test is the difference between before and during the lockdown. A positive value means that the observable has decreased during the lockdown. ***,**,* denote significance at 1%, 5%, 10% level respectively.

Table A.4: Time-variant observables for Italy

	(1)		(2)		T-test
	Before l.d.		During l.d.		Difference
Variable	N	Mean/SE	N	Mean/SE	(1)-(2)
Daily hours of reading	3769	0.950	3769	1.132	-0.182***
		(0.011)		(0.015)	
Daily hours of screen	3769	1.578	3769	3.194	-1.616***
		(0.015)		(0.024)	
Working status of the mother	3769	0.806	3769	0.543	0.263***
		(0.006)		(0.008)	
Working status of the father	3769	0.890	3769	0.681	0.209***
		(0.005)		(0.008)	

Descriptive statistics are based on data collected through online survey during the Covid19 pandemic. Data collection was from 21 April to 11 May for France, and from 7 April to 11 May for Italy. "Daily hours of reading" is the number of hours spent in reading or listening relatives reading. "Daily hours of screen" is the number of hours spent in front of passive screen as TV, Internet, social network etc. "Working status" is a dummy equal to one when the household member declares (or is declared) working in the period (including smartworking during the lockdown).
The T-test is the difference between before and during the lockdown. A positive value means that the observable has decreased during the lockdown. ***,**,* denote significance at 1%, 5%, 10% level respectively.

Differences in children's characteristics

For all of the ttest provided below, we compare children's characteristics between those following online lectures (T=1) and the others (T=0). For the time-variant observables, only period before the lockdown is retained.

Table A.5: French sample before lockdown

	(0)		(2)	T-test	
	Others Methods		Online Lectures		Difference
Variable	N	Mean/SE	N	Mean/SE	(1)-(2)
Daily hours of reading	1900	0.936	1283	0.926	0.009
		(0.016)		(0.020)	
Daily hours of passive screen	1900	0.925	1283	1.255	-0.330***
		(0.017)		(0.024)	
Mother is working	1900	0.838	1283	0.856	-0.017
		(0.008)		(0.010)	
Father is working	1900	0.836	1283	0.810	0.026*
		(0.009)		(0.011)	
Mother works as essential worker[a]	1900	0.209	1283	0.205	0.004
		(0.009)		(0.011)	
Father works as essential worker[a]	1900	0.384	1283	0.357	0.027
		(0.011)		(0.013)	
Having siblings (=1)	1900	0.829	1283	0.776	0.053***
		(0.009)		(0.012)	
University Degree (Mother)	1881	0.553	1268	0.604	-0.051***
		(0.011)		(0.014)	
University Degree (Father)	1699	0.407	1114	0.393	0.014
		(0.012)		(0.015)	

Notes: [a] Essential workers are here categorized as workers who continue to work outside in specific fields, as health, large detailers, building industry.
The value displayed for t-tests are the differences in the means across the groups. ***, **, and * indicate significance at the 1, 5, and 10 percent critical level.

Table A.6: Italian sample before lockdown

	(0)		(1)		T-test
	Other Methods		Online Lectures		Difference
Variable	N	Mean/SE	N	Mean/SE	(1)-(2)
Daily hours of reading	1376	0.956	2393	0.947	0.009
		(0.017)		(0.015)	
Daily hours of passive screen	1376	1.468	2393	1.642	-0.174***
		(0.023)		(0.019)	
Mother is working	1376	0.796	2393	0.812	-0.016
		(0.011)		(0.008)	
Father is working	1376	0.911	2393	0.878	0.034***
		(0.008)		(0.007)	
Mother works as essential worker[a]	1376	0.236	2383	0.221	0.015
		(0.011)		(0.008)	
Father works as essential worker[a]	1288	0.479	2194	0.472	0.007
		(0.014)		(0.011)	
Having siblings (=1)	1376	0.742	2393	0.739	0.003
		(0.012)		(0.009)	
University Degree (Mother)	1366	0.587	2379	0.557	0.031*
		(0.013)		(0.010)	
University Degree (Father)	1281	0.376	2185	0.359	0.017
		(0.014)		(0.010)	

Notes: [a] Essential workers are here categorized as workers who continue to work outside in specific fields, as health, large detailers, building industry.
The value displayed for t-tests are the differences in the means across the groups. ***, **, and * indicate significance at the 1, 5, and 10 percent critical level.

A.3 Heterogeneity analysis with children and family characteristics - Learning Progress

Table A.7: Heterogeneity analysis with children and family characteristics - Learning progress [France]

	Baseline		Boys		Girls		No Siblings		Siblings		Univ.		No Univ.	
		DLM		DLM		DLM		DLM		DLM		DLM		DLM
	(1)	(2)	(3)	(4)	(5)	(6)	(7)	(8)	(9)	(10)	(11)	(12)	(13	(14)
Lockdown	-1.435***		-1.445***		-1.420***		-1.400***		-1.450***		-1.349***		-1.514***	
	(0.049)		(0.070)		(0.058)		(0.087)		(0.054)		(0.059)		(0.078)	
Homework (Ref.)		-1.462***		-1.439***		-1.485***		-1.384***		-1.485***		-1.364***		-1.528***
· Lockdown		(0.051)		(0.068)		(0.050)		(0.081)		(0.054)		(0.066)		(0.092)
Online Lectures		0.069		-0.017		0.155***		-0.033		0.093*		0.037		0.042
· Lockdown		(0.048)		(0.065)		(0.047)		(0.150)		(0.048)		(0.048)		(0.088)
N	6,316	6,316	3,222	3,222	3,094	3,094	1,216	1,216	5,100	5,100	3,584	3,584	1,926	1,926
Within R-Squared	0.698	0.698	0.705	0.705	0.692	0.694	0.721	0.721	0.693	0.694	0.680	0.680	0.721	0.721

All results were estimated using first-difference models on original datasets from Italian and French 2020 Covid19 online surveys. "Lockdown" is a dummy variable equal to one for the period during the school closures and zero before. Parental evaluation of the children's learning progress is defined in the Section 3.1 and standardized in the estimates with a mean of zero and a standard error of one. "Homework" is a dummy equal to one for children benefited from pedagogical contents without interactions with their teachers during the lockdown. "Online Lectures" is a dummy equal to one for children benefited from online interactive lessons during the lockdown. In these estimates, all retained children followed either Homework, or Online Lessons. Therefore, "$Homework \cdot Lockdown$" is purely similar to the "$Lockdown$" term, constituent of the interactive variable "$OnlineLectures \cdot Lockdown$". Coefficient in front of the interactive variable "$OnlineLectures \cdot Lockdown$" must be interpreted as a differential effect from the category of reference, "$Homework \cdot Lockdown$".
Each specification controls for a set of time-variant covariates as working status of the mother and the father, children's time-use in reading and in front of passive screen.
"Sibling" are estimates on subsamples of children having at least one brother or sister at home during the pandemic. "No Siblings" are on only-children. "Univ." are estimates on subsamples of children with at least one parent graduated from University degree. "No Univ." are on children with both parents without an University degree.
Each specification also controls for child individual fixed effects. Standards Errors in parentheses are clustered at region level. ***,**,* indicate significance at 1%, 5%, 10% level respectively.

Table A.8: Heterogeneity analysis with children and family characteristics - Learning progress [Italy]

	Baseline		Boys		Girls		No Siblings		Siblings		Univ.		No Univ.	
		DLM		DLM		DLM		DLM		DLM		DLM		DLM
	(1)	(2)	(3)	(4)	(5)	(6)	(7)	(8)	(9)	(10)	(11)	(12)	(13	(14)
Lockdown	-1.455***		-1.540***		-1.368***		-1.522***		-1.433***		-1.416***		-1.465***	
	(0.058)		(0.074)		(0.054)		(0.071)		(0.062)		(0.078)		(0.063)	
Homework (Ref.)		-1.805***		-1.869***		-1.736***		-1.854***		-1.790***		-1.804***		-1.748***
· Lockdown		(0.082)		(0.100)		(0.084)		(0.090)		(0.102)		(0.110)		(0.085)
Online Lectures		0.482***		0.455***		0.502***		0.451***		0.494***		0.543***		0.383***
· Lockdown		(0.055)		(0.055)		(0.084)		(0.071)		(0.075)		(0.084)		(0.061)
N	6,692	6,692	3,386	3,386	3,306	3,306	1,722	1,722	4,970	4,970	3,840	3,840	2,272	2,272
Within R-Squared	0.793	0.809	0.810	0.824	0.778	0.797	0.800	0.813	0.791	0.809	0.789	0.810	0.799	0.810

All results were estimated using first-difference models on original datasets from Italian and French 2020 Covid19 online surveys. "Lockdown" is a dummy variable equal to one for the period during the school closures and zero before. Parental evaluation of the children's learning progress is defined in the Section 3.1 and standardized in the estimates with a mean of zero and a standard error of one. "Homework" is a dummy equal to one for children benefited from pedagogical contents without interactions with their teachers during the lockdown. "Online Lectures" is a dummy equal to one for children benefited from online interactive lessons during the lockdown. In these estimates, all retained children followed either Homework, or Online Lessons. Therefore, "$Homework \cdot Lockdown$" is purely similar to the "$Lockdown$" term, constituent of the interactive variable "$OnlineLectures \cdot Lockdown$". Coefficient in front of the interactive variable "$OnlineLectures \cdot Lockdown$" must be interpreted as a differential effect from the category of reference, "$Homework \cdot Lockdown$".
Each specification controls for a set of time-variant covariates as working status of the mother and the father, children's time-use in reading and in front of passive screen.
"Sibling" are estimates on subsamples of children having at least one brother or sister at home during the pandemic. "No Siblings" are on only-children. "Univ." are estimates on subsamples of children with at least one parent graduated from University degree. "No Univ." are on children with both parents without an University degree.
Each specification also controls for child individual fixed effects. Standards Errors in parentheses are clustered at region level. ***,**,* denote significance at 1%, 5%, 10% level respectively.

A.4 Heterogeneity analysis with children and family characteristics - Emotional Status

Table A.9: Heterogeneity analysis with children and family characteristics - Emotional Status [France]

	Baseline		Boys		Girls		No Siblings		Siblings		Univ.		No Univ.	
		DLM		DLM		DLM		DLM		DLM		DLM		DLM
	(1)	(2)	(3)	(4)	(5)	(6)	(7)	(8)	(9)	(10)	(11)	(12)	(13	(14)
Lockdown	-0.379***		-0.376***		-0.379***		-0.236		-0.426***		-0.329***		-0.459**	
	(0.066)		(0.100)		(0.094)		(0.138)		(0.063)		(0.064)		(0.158)	
Homework (Ref.)		-0.441***		-0.459***		-0.418***		-0.333*		-0.479***		-0.380***		-0.536**
· Lockdown		(0.068)		(0.108)		(0.088)		(0.154)		(0.061)		(0.045)		(0.175)
Online Lectures		0.158*		0.223*		0.093		0.202		0.144**		0.127		0.233
· Lockdown		(0.074)		(0.121)		(0.119)		(0.183)		(0.066)		(0.072)		(0.176)
N	6,316	6,316	3,222	3,222	3,094	3,094	1,216	1,216	5,100	5,100	3,584	3,584	1,926	1,926
Within R-Squared	0.129	0.131	0.112	0.117	0.153	0.155	0.118	0.123	0.134	0.136	0.114	0.116	0.166	0.172

All results were estimated using first-difference models on original datasets from Italian and French 2020 Covid19 online surveys. "Lockdown" is a dummy variable equal to one for the period during the school closures and zero before. Parental's evaluation of the children's learning progress is defined in the Section 3.1 and standardized in the estimates with a mean of zero and a standard error of one. "Homework" is a dummy equal to one for children benefited from pedagogical contents without interactions with their teachers during the lockdown. "Online Lectures" is a dummy equal to one for children benefited from online interactive lessons during the lockdown. In these estimates, all retained children followed either Homework, or Online Lessons. Therefore, "$Homework \cdot Lockdown$" is purely similar to the "$Lockdown$" term, constituent of the interactive variable "$OnlineLectures \cdot Lockdown$". Coefficient in front of the interactive variable "$OnlineLectures \cdot Lockdown$" must be interpreted as a differential effect from the category of reference, "$Homework \cdot Lockdown$".
Each specification controls for a set of time-variant covariates as working status of the mother and the father, children's time-use in reading and in front of passive screen.
"Sibling" are estimates on subsamples of children having at least one brother or sister at home during the pandemic. "No Siblings" are on only-children. "Univ." are estimates on subsamples of children with at least one parent graduated from University degree. "No Univ." are on children with both parents without an University degree.
Each specification also controls for child individual fixed effects. Standards Errors in parentheses are clustered at region level. ***,**,* indicate significance at 1%, 5%, 10% level respectively.

Table A.10: Heterogeneity analysis with children and family characteristics - Emotional Status [Italy]

	Baseline		Boys		Girls		No Siblings		Siblings		Univ.		No Univ.	
		DLM		DLM		DLM		DLM		DLM		DLM		DLM
	(1)	(2)	(3)	(4)	(5)	(6)	(7)	(8)	(9)	(10)	(11)	(12)	(13	(14)
Lockdown	-0.796***		-0.895***		-0.691***		-0.839***		-0.781***		-0.727***		-0.852***	
	(0.075)		(0.085)		(0.099)		(0.118)		(0.082)		(0.109)		(0.101)	
Homework (Ref.)		-0.884***		-0.989***		-0.770***		-0.870***		-0.892***		-0.853***		-0.911***
· Lockdown		(0.085)		(0.109)		(0.125)		(0.182)		(0.087)		(0.130)		(0.103)
Online Lectures		0.122*		0.130		0.109		0.042		0.152*		0.177		0.079
· Lockdown		(0.067)		(0.096)		(0.076)		(0.144)		(0.078)		(0.103)		(0.067)
N	6,692	6,692	3,386	3,386	3,306	3,306	1,722	1,722	4,970	4,970	3,840	3,840	2,272	2,272
Within R-Squared	0.344	0.345	0.364	0.365	0.326	0.327	0.378	0.378	0.334	0.336	0.316	0.319	0.397	0.398

All results were estimated using first-difference models on original datasets from Italian and French 2020 Covid19 online surveys. "Lockdown" is a dummy variable equal to one for the period during the school closures and zero before. Parental's evaluation of the children's learning progress is defined in the Section 3.1 and standardized in the estimates with a mean of zero and a standard error of one. "Homework" is a dummy equal to one for children benefited from pedagogical contents without interactions with their teachers during the lockdown. "Online Lectures" is a dummy equal to one for children benefited from online interactive lessons during the lockdown. In these estimates, all retained children followed either Homework, or Online Lessons. Therefore, "$Homework \cdot Lockdown$" is purely similar to the "$Lockdown$" term, constituent of the interactive variable "$OnlineLectures \cdot Lockdown$". Coefficient in front of the interactive variable "$OnlineLectures \cdot Lockdown$" must be interpreted as a differential effect from the category of reference, "$Homework \cdot Lockdown$".
Each specification controls for a set of time-variant covariates as working status of the mother and the father, children's time-use in reading and in front of passive screen.
"Sibling" are estimates on subsamples of children having at least one brother or sister at home during the pandemic. "No Siblings" are on only-children. "Univ." are estimates on subsamples of children with at least one parent graduated from University degree. "No Univ." are on children with both parents without an University degree.
Each specification also controls for child individual fixed effects. Standards Errors in parentheses are clustered at region level. ***,**,* indicate significance at 1%, 5%, 10% level respectively.
