# Supplementary material for: Child development and distance learning in the age of COVID-19
Source: Rev Econ Househ. 2022 Apr 5;20(3):659–85. doi: 10.1007/s11150-022-09606-w (PMC8982654; doi:10.1007/s11150-022-09606-w)
Supplement: Supplementary file 2 — French questionnaire_EN [file 11150_2022_9606_MOESM2_ESM.pdf]

Household survey on the socio-economic impact of Covid-19

Following the Covid-19 pandemic, the confinement and closure of schools represent a significant challenge for families, particularly in terms of reconciling work and family life and the educational follow-up of children.
In this regard, our team of researchers from the *Centre d'Etudes et de Recherche sur le Développement International* (CERDI) of the University of Clermont Auvergne is conducting a survey to collect information on the impact of the sanitary crisis and the confinement on the family organization. Your participation in our study is precious and we thank you in advance for the time you will devote to us.
The survey takes between 5 and 15 minutes and participation is voluntary.
We will collect survey data until May 10.

Francesca Marchetta and Hugues Champeaux, with the collaboration of Lidia Farré (University of Barcelona), Libertad González (Universitat Pompeu Fabra) and Lucia Mangiavacchi (Department of Political Science of the University of Perugia).
*Mandatory

1. Based on the following information, I agree to participate in the survey described above, and I give my consent for my data to be used for the for the purpose described: *

Only one possible answer

- ⬭ Yes *Proceed to question 2*
- ⬭ No

2. INFORMATION - PROTECTION OF PERSONAL DATA PERSONAL DATA

In accordance with the regulations on the protection of personal data in force, and as mentioned above, the purpose of this survey is to study the impact of health emergencies and confinement on family organization and is voluntary. The controller is the UCA. The data processed in this questionnaire correspond to the living habits during and before the confinement of household's resident in France. The processed data come only from your answers to the questions. The personal data collected in the framework of this questionnaire will only be handled by the researchers involved in the study and will be kept for two years. They will then be archived on paper or computer for a period in accordance with the regulations in force. No data will be transferred outside the European Union. The personal data collected in the context of this questionnaire will be used in a confidential manner and at an aggregate level. Concerning the exercise of your rights, you can consult the CNIL web page "Understanding your rights on personal data". Exercising these rights: The University's Data Protection Officer (DPO) is your contact for any request to exercise your rights regarding this processing: - By e-mail: dpo@uca.fr - By post: DAJI - DPO _ Université Clermont Auvergne _ 49, boulevard François Mitterrand - CS 60032 _ 63001 Clermont-Ferrand Cedex 1. Complaint to the CNIL: If you feel, after having contacted us, that your rights have not been respected, you can lodge a complaint with the CNIL.

Several answers possible

☐ Click on "Next" below to start the questionnaire

Individual information

3. Gender:

- Man
- Woman

4. Year of birth:

____

5. Country of birth:

Only one possible answer

- France
- Foreign

6. What is your postal code?

____

7. What is your educational level?

- Brevet
- CAP, BEP
- Bac, Bac+2 (BTS, DEUG)
- Bac+3 or more (Bachelor, Master’s, PhD)
- No qualification

8. How many people live in the household? We refer to the period since lockdown.

____

9. What is your current degree of isolation?

Only one possible answer

- Total, I don't go out because I'm in quarantine
- Confined, I go out to cover basic needs (shopping, going to the doctor, walking the dog, doing sports)
- Partial, I continued to go out to work

10. What is your socio-professional category (SPC)?

Only one possible answer

- Farmers/agriculturists
- Craftsmen, traders, and entrepreneurs
- Executives and higher intellectual professions (liberal professions, engineers and technical, administrative, and commercial executives, scientific professions)
- Intermediate professions (schoolteachers, administrative staff, intermediate occupations in health and social work, technicians, supervisors)
- Employees (civil servants, police, military, commercial employees, direct services to individuals)
- Workers (skilled and unskilled workers, agricultural workers, drivers)

Previous work situation

11. What was your employment status before the period of lockdown?

Only one possible answer

- Full-time employee
- Part-time employee
- Full-time self-employed — *Skip to question 13*
- Part-time self-employed — *Skip to question 13*
- I didn't have a job, but I was searching — *Skip to question 22*
- I didn't have a job and I wasn't searching for one — *Skip to question 22*
- I was on leave — *Skip to question 22*
- I didn't have a job and took care of the home and family — *Skip to question 22*
- Student — *Skip to question 22*
- Retired — *Skip to question 22*
- Other: — *Skip to question 22*

12. What kind of contract did you have?

Only one possible answer

- Permanent
- Fixed term

13. In which sector did you work?

Only one possible answer

- Healthcare
- Leisure: hotels, restaurants, tourism, etc.
- Essential services: food, livestock, fishing, agriculture, transport, deliveries, etc.
- Teaching or research
- Construction jobs
- Industrial manufacturing
- Other:

14. How many hours did you work per week?

____

15. What percentage of these hours were you working from home (tele-working or smart working)?

- Zero
- A few hours, less than 33%
- Between 33% and 66%
- More than 66%
- 100%

16. Since the lockdown period, have you experienced a loss of income?

Only one possible answer

- Yes
- No

17. What is your current working situation?

Only one possible answer

- ⬭ I have the same job, with the same income conditions as before the lockdown
- ⬭ I have the same job, but with different income conditions
- ⬭ I changed job
- ⬭ I am not currently working (activity suspension, unemployment) *Skip to question 22*
- ⬭ I have lost my job and I'm looking to find another one *Skip to question 22*

18. Since the lockdown period, how many hours do you work per week?

____

19. What percentage of these hours were you working from home (tele-working or smart working)?

Only one possible answer

- ⬭ Zero
- ⬭ A few hours, less than 33%
- ⬭ Between 33% and 66%
- ⬭ More than 66%
- ⬭ 100%

20. More specifically, how many hours of tele-working are you currently doing?

____

21. On a 0 to 10 scale, how likely are you to lose your job over the next year?

	0	1	2	3	4	5	6	7	8	9	10	
I will certainly keep my job	⬭	⬭	⬭	⬭	⬭	⬭	⬭	⬭	⬭	⬭	⬭	I will certainly lose my job

22. What is the composition of your household?

Only one possible answer

- ⬭ Couple with or without children
- ⬭ Single parent family *Skip to question 43*
- ⬭ Single without children *Skip to question 147*

Partner

23. Gender of your partner

Only one possible answer

- ⬭ Man
- ⬭ Women

24. What is your spouse/partner's year of birth?

____

25. What is her/his country of birth?

Only one possible answer

- ⬭ France
- ⬭ Foreign country

26. What is her/his educational level?

- Brevet
- CAP, BEP
- Bac, Bac+2 (BTS, DEUG)
- Bac+3 or more (Bachelor, Master's, PhD)
- No qualification

27. What is her/his current degree of isolation?

Only one possible answer

- Total, she/he does not go out because I'm in quarantine
- Confined, she/he goes out to cover basic needs (shopping, going to the doctor, walking the dog, doing sports)
- Partial, she/he continued to go out to work

28. Of all monthly income of the household, what share does your spouse/partner contribute to? Think about all income sources (salary, unemployment benefit, pension, rents, etc.)

Only one possible answer

- She/he does not contribute financially
- Less than 25%
- More than 25% but less than 50%
- About half 50% as my contribution
- More than 50% but less than 75%
- More than 75% but less than 90%
- She/he brings in all of family income

29. What is her/his socio-professional category (SPC)?

Only one possible answer

- Farmers/agriculturists
- Craftsmen, traders, and entrepreneurs
- Executives and higher intellectual professions (liberal professions, engineers and technical, administrative, and commercial executives, scientific professions)
- Intermediate professions (schoolteachers, administrative staff, intermediate occupations in health and social work, technicians, supervisors)
- Employees (civil servants, police, military, commercial employees, direct services to individuals)
- Workers (skilled and unskilled workers, agricultural workers, drivers)

30. Before lockdown period, what was your spouse/partner's working situation?

Only one possible answer

- Full-time employee
- Part-time employee
- Full-time self-employed — Skip *to question 32*
- Part-time self-employed — Skip *to question 32*
- I didn't have a job, but I was searching — *Skip to question 41*
- I didn't have a job and I wasn't searching for one — *Skip to question 41*
- I was on leave — Skip *to question 41*
- I didn't have a job and took care of the home and family — *Skip to question 41*
- Student — *Skip to question 41*
- Retired — *Skip to question 41*
- Other:______________ — *Skip to question 41*

Previous work situation of spouse/partner

31. Before lockdown period, what kind of contract did your spouse/partner have?

- Permanent
- Fixed term

32. Before lockdown period, in which sector did she/he worked?

Only one possible answer

- Healthcare
- Leisure: hotels, restaurants, tourism, etc.
- Essential services: food, livestock, fishing, agriculture, transport, deliveries, etc.
- Teaching or research
- Construction jobs
- Industrial manufacturing
- Other:

33. Before lockdown period, how many hours a week did your spouse/partner work?

____

34. What percentage of these hours were he/she was working from home (tele-working or smart working)?

Only one possible answer

- Zero
- A few hours, less than 33%
- Between 33% and 66%
- More than 66%
- 100%

Current work situation of spouse/partner

35. Since the lockdown period, has your spouse/partner experienced a loss of income?

Only one possible answer

- Yes
- No

36. What is your spouse/partner's current employment status?

Only one possible answer

- He/she has the same job, with the same income conditions as before the lockdown
- He/she has the same job, but with different income conditions
- He/she changed jobs
- He/she is not currently working (activity suspension, unemployment) *Skip to question 41*
- He/she have lost her/his job and is looking to find another one *Skip to question 41*

37. Since lockdown period, how many hours did he/she work per week?

____

38. What percentage of these hours were he/she working from home (tele-working or smart working)?

Only one possible answer

- Zero
- A few hours, less than 33%
- Between 33% and 66%
- More than 66%
- 100%

39. More specifically, how many hours per week does your spouse/partner currently spend on teleworking?

____

40. On a 0 to 10 scale, how likely is your partner to lose her/his job over the next year?

	0	1	2	3	4	5	6	7	8	9	10	
Certainly keep her/his job.												Certainly lose her/his job

41. Since confinement, have you experienced conflict situations with your spouse/partner?

Only one possible answer

- Much more than before the lockdown
- A little bit more than before the lockdown
- I don't feel any differences
- A little bit less than before the lockdown
- Much less than before the lockdown

Family Status

42. How many children under age 16 live with you and your spouse/partner?

Only one possible answer

- None *Skip to question 143*
- 1 *Skip to question 44*
- 2 *Skip to question 44*
- 3 *Skip to question 44*
- 4 *Skip to question 44*
- More than 4 *Skip to question 44*

43. How many children under age 16 live with you?

Only one possible answer

- None *Skip to question 147*
- 1 *Skip to question 55*
- 2 *Skip to question 55*
- 3 *Skip to question 55*
- 4 *Skip to question 55*
- More than 4 *Skip to question 55*

44. Before the lockdown period, how did you split the following tasks with your spouse/partner?

Only one possible answer per raw

	Always me	Mostly me	Equally	Mostly spouse/partner	Always spouse/partner	Another person
Shopping						
Laundry						
Cooking						
Housekeeping						
Follow children with homework or educational activities						
Playing with children						

45. Before the lockdown period, on average, how many hours a week did you spend cooking, cleaning your home or taking care of laundry?

We are talking about hours on average PER week. Answer only for the respondent.

____

46. Currently, how do you split the following tasks with your spouse/partner?

CAREFUL: we are talking here about hours per week OUTSIDE SCHOOL HOLIDAYS.

Only one possible answer per raw

	Always me	Mostly me	Equally	Mostly spouse/partner	Always spouse/partner	Another person
Shopping						
Laundry						
Cooking						
Housekeeping						
Follow children with homework or educational activities						
Playing with children						

47. Since the lockdown period, on average, how many hours a week do you spend cooking, cleaning your home or taking care of laundry?

We are talking about hours on average PER week. Answer only for the respondent.

____

48. Are the children living with you your biological children?

Only one possible answer

- Yes, all
- Yes, some of them
- No

49. Are the children living with you biological children of your spouse/partner?

Only one possible answer

- Yes, all
- Yes, some of them
- No

50. How many children in the household are in a joint custody?

____

51. Before the lockdown, how many hours a week were you spending doing homework or educational activities with your children?

CAREFUL: we are talking here about hours per week OUTSIDE SCHOOL HOLIDAYS!

____

52. Currently, how many hours a week do you spend doing homework or educational activities with your children?

CAREFUL: we are talking here about hours per week OUTSIDE SCHOOL HOLIDAYS!

____

53. Before the lockdown, how many hours a week was your spouse/partner spending doing homework or educational activities with your children?

CAREFUL: we are talking here about hours per week OUTSIDE SCHOOL HOLIDAYS!

____

54. Currently, how many hours per week does your spouse/partner spend doing homework or doing educational activities with children?

CAREFUL: we are talking here about hours per week OUTSIDE SCHOOL HOLIDAYS!

____

Skip to question 58

55. How many of your children are in a joint custody?

____

56. Before the lockdown, how many hours a week were you spending doing homework or educational activities with your children?

CAREFUL: we are talking here about hours per week.

____

57. Currently, how many hours a week do you spend doing homework or educational activities with your children?

CAREFUL: we are talking here about hours per week OUTSIDE SCHOOL HOLIDAYS!

____

Children (repeated for each child)

58. Year of birth

____

59. Month of birth

Only one possible answer

- January
- February
- March
- April
- May
- June
- July
- August
- September
- October
- November
- December

60. Gender

Only one possible answer

- Male
- Female

61. Select the school in which she/he is currently enrolled:

- Nursery
- Kindergarten: 1st year
- Kindergarten: 2nd year
- Kindergarten: 3rd year
- Primary school: 1st year
- Primary school: 2nd year
- Primary school: 3rd year
- Primary school: 4th year
- Primary school: 5th year
- Middle school: 1st year
- Middle school: 2nd year
- Middle school: 3rd year
- Middle school: 4th year
- High school: 1st year
- High school: 2nd year
- High school: 3rd year
- In a specialized class (handicap or other)
- She/he is not enrolled at school

62. If your working sector is health, how many hours a day does your child spend at school or recreation center since the lockdown?

We are talking here about hours per week OUTSIDE SCHOOL HOLIDAYS!

Only one possible answer

- ◯ No time at all
- ◯ 1-2 hour on average
- ◯ 2-4 hour on average
- ◯ 4-6 hour on average
- ◯ More than 6 hours on average
- ◯ I don’t work in health sector

63. How many hours a day does your child spend studying since the lockdown?

We are referring to a typical school day in the period outside of your child's spring school holidays

Only one possible answer

- ◯ No time at all
- ◯ Less than 1 hour
- ◯ 1-2 hour on average
- ◯ 2-4 hour on average
- ◯ 4-6 hour on average
- ◯ More than 6 hours

64. Check the modalities used by his/her teachers for distance learning monitoring, since lockdown

Several answers are possible.

- ☐ Online classes (live video)
- ☐ Sending lessons, exercises and/or activities by e-mail
- ☐ Sending videos with educational content created by the teacher
- ☐ Sending videos with educational content created by others
- ☐ Chat with teachers and other students
- ☐ Other
- ☐ None

65. What tools do you have to enable your child to follow distance learning?

Several answers are possible.

	Personal (child's one)	*Shared (by family or owned by parents)*	*None*
Computer	☐	☐	☐
Digital tablet	☐	☐	☐
Smartphone	☐	☐	☐

66. How would you rate her/his current learning progress from 0 to 10?

Do not answer if there are no activities proposed by the teacher.

Only one possible answer

	0	1	2	3	4	5	6	7	8	9	10	
Not progressing at all	○	○	○	○	○	○	○	○	○	○	○	As when attending school

67. Are you experiencing difficulties in helping your child with her/his learning?

Only one possible answer

	0	1	2	3	4	5	6	7	8	9	10	
No difficulties	○	○	○	○	○	○	○	○	○	○	○	A lot of difficulties

68. Before the lockdown, how many hours per school day did your child spend on average:

CAREFUL, we are talking about the number of hours per school day

Only one answer possible per raw

	None	*Less than 1 hour*	*Between 1 and 2 hours*	*Between 2 and 3 hours*	*More than 3 hours*
With the grandparents	○	○	○	○	○
With a babysitter/nanny	○	○	○	○	○
At the recreation center	○	○	○	○	○
In front of screens (TV, internet, video games, youtube)	○	○	○	○	○
To read or listen to books and stories	○	○	○	○	○

69. Since the lockdown, how many hours per school day did your child spend on average:

CAREFUL, we are talking about the number of hours per school day, in the period of lockdown, outside school holidays

Only one answer possible per raw

	None	*Less than 1 hour*	*Between 1 and 2 hours*	*Between 2 and 3 hours*	*More than 3 hours*
With the grandparents	○	○	○	○	○
With a babysitter/nanny	○	○	○	○	○
At the leisure center	○	○	○	○	○
In front of screens (TV, internet, video games, youtube)	○	○	○	○	○
To read or listen to books and stories	○	○	○	○	○

70. Before the lockdown, how many hours a week did she/he spend on extracurricular activities (e.g. music, physical activity, etc.)?

Only one possible answer

- ○ No time at all
- ○ 1 hour on average
- ○ 1-3 hour on average
- ○ 3-4 hour on average
- ○ More than 4 hours

71. Since the lockdown, how many hours a week did she/he spend on extracurricular activities (e.g. music, physical activity, etc.)?

Only one possible answer

- No time at all
- 1 hour on average
- 1-3 hour on average
- 3-4 hour on average
- More than 4 hours

72. How do you judge the evolution of your personal relationship with her/him since school closures?

Only one possible answer

- Significantly worsened
- Slightly worsened
- Unchanged
- Slightly improved
- Much improved

73. How do you judge her/his emotional status after school closures?

Only one possible answer

- Significantly worsened
- Slightly worsened
- Unchanged
- Slightly improved
- Much improved

74. Is there another child?

Only one possible answer

- Yes — *Loop questions 58-74 until "No"*
- No — *Skip to question 147*

143. Before the lockdown period, how did you split the following tasks with your spouse/partner? *
Only one possible answer per raw

	Always me	Mostly me	Equal	Mostly spouse/partner	Always spouse/partner	Another person
Shopping						
Laundry						
Cooking						
Housekeeping						

144. Before the lockdown period, on average, how many hours a week did you spend cooking, cleaning your home or taking care of laundry?

We are talking about hours on average PER week. Answer only for the respondent.

____

145. Currently, how do you split the following tasks with your spouse/partner?

Only one possible answer per raw

	Always me	Mostly me	Equal	Mostly spouse/partner	Always spouse/partner	Another person
Shopping	◯	◯	◯	◯	◯	◯
Laundry	◯	◯	◯	◯	◯	◯
Cooking	◯	◯	◯	◯	◯	◯
Housekeeping	◯	◯	◯	◯	◯	◯

146. Since the lockdown period, on average, how many hours a week would you spend cooking, cleaning your home or taking care of laundry?

We are talking about hours on average PER week. Answer only for the respondent.

____

147. Since the lockdown, do you feel stressed?

Only one possible answer

	0	1	2	3	4	5	6	7	8	9	10	
Not at all	◯	◯	◯	◯	◯	◯	◯	◯	◯	◯	◯	Extremely

148. Since the lockdown, are you able to take care of yourself more than before?

Only one possible answer

- ◯ Yes, I take more care of myself
- ◯ As much as before
- ◯ No, I take less care of myself

149. Do you have regular contacts with your relatives? (Parents or friends, by phone or e-mail)

Only one possible answer

- ◯ Yes, several times a day
- ◯ Once a day
- ◯ Less than once a day
- ◯ Less than three times a week
- ◯ Once a week
- ◯ Less than once a week

We thank you for your time and your patience. We can keep you informed of the results of our investigation, by emailing us directly: Francesca Marchetta (francesca.marchetta@uca.fr); Hugues Champeaux (hugues.champeaux@etu.uca.fr)
