# Supplementary material for: Child development and distance learning in the age of COVID-19
Source: Rev Econ Househ. 2022 Apr 5;20(3):659–85. doi: 10.1007/s11150-022-09606-w (PMC8982654; doi:10.1007/s11150-022-09606-w)
Supplement: Supplementary file 3 — Italian_questionnaire_EN [file 11150_2022_9606_MOESM3_ESM.pdf]

Household survey on the socio-economic impact of Covid-19

General information

1. Gender
   - Man
   - Woman
2. Year of birth
3. Country of birth
   - Italy
   - Foreign
4. In which region do you live?
   - Piedmont
   - Aosta Valley
   - Lombardy
   - Trentino Alto Adige
   - Veneto
   - Friuli Venezia Giulia
   - Liguria
   - Emilia Romagna
   - Tuscany
   - Umbria
   - Brands
   - Lazio
   - Abruzzo
   - Molise
   - Campania
   - Puglia
   - Basilicata
   - Calabria
   - Sicily
   - Sardinia
5. In which province?
6. What is your postal code?
7. Do you have a university degree (bachelor, master's or PhD)?
   - Yes
   - No
8. How many people live in the household?
9. What is your current degree of isolation?
   - Maximum, I am in isolation for COVID-19 or contacts with positive cases
   - Normal, I go out only to cover basic needs (shopping, doctor, take the dog for a walk)
   - Partial, I regularly go out for work, but for a few hours/days
   - Low, I continued to work normally

Previous work situation

10. Before the closure of schools of all levels, due to the Covid-19 lockdown, what was your work situation?

- Full-time employee
- Part-time employee
- Full-time self-employed *Skip to question 12*
- Part-time self-employed *Skip to question 12*
- I didn't have a job but I was searching *skip to question 21*
- I didn't have a job and I wasn't searching for one *skip to question 21*
- I was on leave *skip to question 21*
- I didn't have a job and took care of the home and family *skip to question 21*
- Other (student, pensioner, etc.) *Skip to question 21*

11. Before schools closed, what kind of contract did you have?

- Permanent
- Fixed term

12. Before schools closed, in which industrial sector were you working?

- Healthcare
- Leisure: hotels, restaurants, tourism, etc.
- Essential services: food, livestock, fishing, agriculture, transport, deliveries, etc.
- Other sector

13. Briefly describe your job or occupation:

14. Before schools closed, how many hours a week were you working?

15. What percentage of these hours were you working from home (tele-working or smart-working)?

- Zero
- A few hours, less than 33%
- Between 33% and 66%
- More than 66%

Current work situation

16. What is your current work situation?

- I have the same job, with the same economic conditions and the same working hours as before *Skip to question 18*
- I have the same job, but with different economic conditions or working hours
- I am not currently working (activity suspension, unemployment, “cassa integrazione”, or the like) *Skip to question 20*
- I have lost my job and I will have to find another one *Skip to question 21*

17. To what extent have your working conditions changed? (you can choose multiple options)

- [ ] I work more hours
- [ ] Work less hours
- [ ] Gain more
- [ ] Gain less More
- [ ]

18. How much tele-working are you currently doing?

- The same hours I spent working in presence before the lockdown
- More hours than I spent working in presence before the lockdown
- Fewer hours than I spent working in presence before the lockdown

19. More specifically, how many hours per week of tele-working are you currently doing?

20. On a 0 to 10 scale, how likely are you to lose your job over the next year?

	0	1	2	3	4	5	6	7	8	9	10	
I will certainly keep my job	◯	◯	◯	◯	◯	◯	◯	◯	◯	◯	◯	I will certainly lose my job

21. Are you married or cohabiting?

- ◯ Yes
- ◯ No *Skip to question 39*

Partner

22. What is your spouse/partner's year of birth?

23. What is her/his country of birth?

- ◯ Italy
- ◯ Foreign

24. Does your spouse/partner have a university degree (bachelor, master, PhD)?

- ◯ Yes
- ◯ No

25. What is the current degree of isolation of your spouse/partner?

- ◯ Maximum, she/he is in isolation for COVID-19 or contacts with positive cases
- ◯ Normal, she/he only goes out to cover basic needs (shopping, doctor, take the dog for a walk)
- ◯ Partial, she/he regularly goes out for work, but only for a few hours/days
- ◯ Low, she/he is going out for work normally

26. Of all monthly income of the household, what share does your spouse/partner contribute to? Think about all income sources (salary, unemployment benefit, pension, rents, etc.)

- ◯ She/he does not contribute financially
- ◯ Less than 10%
- ◯ More than 10% but less than 25%
- ◯ More than 25% but less than 50%
- ◯ About half, 50%
- ◯ More than 50% but less than 75%
- ◯ More than 75% but less than 90%
- ◯ Over 90%
- ◯ She/he brings in all of family income

27. Is your spouse/partner the father or mother of your children (at least one)?

- ◯ Yes
- ◯ No

28. Before school closures, what was your spouse/partner's work situation?

- ◯ Full-time employee
- ◯ Part-time employee
- ◯ Full-time self-employed *skip to question 30*
- ◯ Part-time self-employed *skip to question 30*
- ◯ She/he didn't have a job but he was searching for one *skip to question 39*
- ◯ She/he didn't have a job and wasn't searching for one *skip to question 39*
- ◯ She/he was on leave *skip to question 39*
- ◯ She/he didn't have a job and took care of the house and family *skip to question 39*
- ◯ Other (student, pensioner, etc.) *skip to question 39*

Previous work situation of spouse/partner

29. Before schools closed, what kind of contract did your spouse/partner have?

- Permanent
- Fixed term

30. Before schools closed, in which industrial sector did your spouse/partner work?

- Healthcare
- Leisure: hotels, restaurants, tourism, etc.
- Essential services: food, livestock, fishing, agriculture, transport, deliveries, etc.
- Other sectors

31. Briefly describe the work or employment of your spouse/partner:

32. Before schools closed, how many hours a week did your spouse/partner work?

33. What percentage of these hours did your spouse/partner work from home (tele-working or smart-working)?

- Zero
- A few hours, less than 33%
- Between 33% and 66%
- Over 66%

Current work situation of spouse/partner

34. What is your spouse/partner's current work situation?

- She/he has the same work, with the same economic conditions and working hours — *Skip to question 36*
- She/he has the same work, but with different economic conditions or working hours
- She/he is not currently working (activity suspension, unemployment, "cassa integrazione", or the like) — *Skip to question 38*
- She/he has lost his job and will have to find another — *Skip to question 39*

35. To what extent have her/his working conditions changed since before the closure of schools? (you can choose multiple options)

- [ ] Works more hours
- [ ] Works fewer hours
- [ ] Earns more
- [ ] Earns less More
- [ ]

36. How much tele-working is your spouse/partner currently doing?

- The same hours she/he spent working in presence before the lockdown
- More hours than she/he spent working in presence before the lockdown
- Fewer hours than she/he spent working in presence before the lockdown

37. More specifically, how many hours per week does your spouse/partner currently spend on teleworking?

38. On a 0 to 10 scale, how likely is your partner to lose her/his job over the next year?

	0	1	2	3	4	5	6	7	8	9	10	
Certainly keep her/his job.	○	○	○	○	○	○	○	○	○	○	○	Certainly lose her/his job

Children younger than 16

39. How many children under age 16 live with you?

- None *Skip to question 121*
- 1
- 2
- 3
- 4
- More than 4

40. Before school closed, how did you split the following tasks with your spouse/partner?

	All me	Mostly me	Equal	Mostly spouse/partner	Always spouse/partner	Another person
Shopping						
Laundry						
Cooking						
Housekeeping						
Follow children with homework or educational activities						
Playing with children						

41. Currently, how do you split the following tasks with your spouse/partner?

	All me	Mostly me	Equal	Mostly spouse/partner	Always spouse/partner	Another person
Shopping						
Laundry						
Cooking						
Housekeeping						
Follow children with homework or educational activities						
Playing with children						

42. Before schools closed, how many hours a week were you spending doing homework or educational activities with your children?

43. Currently, how many hours a week do you spend doing homework or educational activities with your children?

44. Before school closed, how many hours a week did your spouse/partner spend doing homework or educational activities with children?

45. Currently, how many hours per week does your spouse/partner spend doing homework or doing educational activities with children?

46. Before school closed, how many hours a day your children spent with their grandparents or other cohabiting family members (if any)?

47. Currently, how many hours a day do your children spend with grandparents or other cohabiting family members (if any)?

48. Before schools closed, how many hours a day did your children spend with a babysitter?

49. Currently, how many hours a day do your children spend with a babysitter?

Children (repeated for each child)

50. Year of birth

51. Month of birth

- January
- February
- March
- April
- May
- June
- July
- August
- September
- October
- November
- December

52. Gender

- Male
- Female

53. Select the school to which she/he is currently enrolled:

- Nursery *Skip-to-question 59*
- Kindergarten
- First Year Primary
- Second Year Primary
- Third Year Primary
- Fourth Year Primary
- Fifth Year Primary
- First year Secondary (1st level – "scuole medie")
- Second year Secondary (1st level – "scuole medie")
- Third year Secondary (1st level – "scuole medie")
- First secondary year (2nd level - High school)
- Second year Secondary (2nd level - High school)
- Third year Secondary (2nd level - High school)
- He is not enrolled in any school *Skip to question 59*

54. Do her/his teachers offer online classes?

- Yes
- No

55. Select the teaching mode offered by her/his school after closure

- Live online classis, assigned tasks and individual study
- Only assigned tasks and individual study
- She/he is not participating to any teaching activities

56. Do you have a computer or tablet that she/he can use for online teaching tasks?

- Yes
- No

57. How many hours of online classes does she/he follow on average per day?

58. How would you rate her/his current learning progress from 0 to 10?

	0	1	2	3	4	5	6	7	8	9	10	
Not progressing at all												As when attending school

59. Before the closure of schools, how many hours a week did she/he spend on extracurricular activities (e.g. music, physical activity, etc.)?

60. Currently, how many hours a week does she/he spend on distance extracurricular activities (e.g. music, physical activity, etc.)?

61. Before school closed, how many hours a day did she/he watch television, play video games, pass on social networks (istagram, tiktok and similar), watch videos on the internet (netflix, youtube, etc.)?

62. Currently, how many hours a day does she/he watch television, play video games, pass on social networks (histagram, tiktok and similar), watch videos on the internet (netflix, youtube, etc.)?

63. Before schools closed, how many hours a day did she/he read (or listen to you read) books or stories?

64. Currently, how many hours a day does she/he read (or hear you read) books or stories?

65. How do you judge the evolution of your personal relationship with her/him since school closures?

- Significantly worsened
- Slightly worse
- Unchanged
- Slightly improved
- Much improved

66. How do you judge her/his emotional status after school closures?

- Significantly worsened
- Slightly worse
- Unchanged
- Slightly improved
- Much improved

67. Is there another child?

- Yes — *Loop questions 50-67 until "No"*
- No — *Skip to section 33 (Thank you so much for your collaboration and for your time..)*
